# Quantitative computed tomography–derived clusters: Redefining airway remodeling in asthmatic patients^☆^

**DOI:** 10.1016/j.jaci.2013.09.039

**Published:** 2014-03

**Authors:** Sumit Gupta, Ruth Hartley, Umair T. Khan, Amisha Singapuri, Beverly Hargadon, William Monteiro, Ian D. Pavord, Ana R. Sousa, Richard P. Marshall, Deepak Subramanian, David Parr, James J. Entwisle, Salman Siddiqui, Vimal Raj, Christopher E. Brightling

**Affiliations:** a Department of Infection, Inflammation and Immunity, Institute for Lung Health, University of Leicester, Leicester, United Kingdom; b Radiology Department, Glenfield Hospital, University Hospitals of Leicester NHS Trust, Leicester, United Kingdom; c Respiratory Therapy Unit, GlaxoSmithKline, Stockley Park, Uxbridge, United Kingdom; d Department of Respiratory Medicine, University Hospitals Coventry and Warwickshire, Coventry, United Kingdom; e Radiology Department, Wellington Hospital, Capital and Coast District Health Board, Wellington, New Zealand

**Keywords:** Asthma, airway remodeling, distal airway, CT, quantitative imaging, phenotypes, cluster analysis, fractal analysis, ATS, American Thoracic Society, BSA, Body surface area, CT, Computed tomography, D_av_, Averaged fractal dimension, D_e_, Most efficient cover fractal dimension, D_sc_, Slope-corrected fractal dimension, D_sce_, Slope-corrected most-efficient covering fractal dimension, FRC, Functional residual capacity, HU, Hounsfield units, ICC, Intraclass correlation coefficient, LA, Lumen area, LV, Lumen volume, MLD E/I, Mean lung density expiratory/inspiratory ratio, Pi10, Hypothetical airway with internal perimeter of 10 mm, Po20, Hypothetical airways with outer airway perimeter of 20 mm, RB1, Right upper lobe apical segmental bronchus, ROI, Region of interest, RV, Residual volume, TLC, Total lung capacity, VI, Voxel index, VI_−850_ E-I, VI−850 change on paired inspiratory and expiratory CT scan, VI_−850/−950_ E-I, Voxel index change of percent voxels between −950 and −850 HU on paired inspiratory and expiratory CT scan, WA, Wall area, WV, Wall volume

## Abstract

**Background:**

Asthma heterogeneity is multidimensional and requires additional tools to unravel its complexity. Computed tomography (CT)–assessed proximal airway remodeling and air trapping in asthmatic patients might provide new insights into underlying disease mechanisms.

**Objectives:**

The aim of this study was to explore novel, quantitative, CT-determined asthma phenotypes.

**Methods:**

Sixty-five asthmatic patients and 30 healthy subjects underwent detailed clinical, physiologic characterization and quantitative CT analysis. Factor and cluster analysis techniques were used to determine 3 novel, quantitative, CT-based asthma phenotypes.

**Results:**

Patients with severe and mild-to-moderate asthma demonstrated smaller mean right upper lobe apical segmental bronchus (RB1) lumen volume (LV) in comparison with healthy control subjects (272.3 mm^3^ [SD, 112.6 mm^3^], 259.0 mm^3^ [SD, 53.3 mm^3^], 366.4 mm^3^ [SD, 195.3 mm^3^], respectively; *P* = .007) but no difference in RB1 wall volume (WV). Air trapping measured based on mean lung density expiratory/inspiratory ratio was greater in patients with severe and mild-to-moderate asthma compared with that seen in healthy control subjects (0.861 [SD, 0.05)], 0.866 [SD, 0.07], and 0.830 [SD, 0.06], respectively; *P* = .04). The fractal dimension of the segmented airway tree was less in asthmatic patients compared with that seen in control subjects (*P* = .007). Three novel, quantitative, CT-based asthma clusters were identified, all of which demonstrated air trapping. Cluster 1 demonstrates increased RB1 WV and RB1 LV but decreased RB1 percentage WV. On the contrary, cluster 3 subjects have the smallest RB1 WV and LV values but the highest RB1 percentage WV values. There is a lack of proximal airway remodeling in cluster 2 subjects.

**Conclusions:**

Quantitative CT analysis provides a new perspective in asthma phenotyping, which might prove useful in patient selection for novel therapies.

Asthma remains a major health care burden affecting an estimated population of 300 million persons worldwide, with an annual premature fatality of 250,000 persons.^1^ Approximately 5% to 10% of patients have severe asthma and do not respond adequately to traditional treatment. These patients have severely impaired quality of life and impose a disproportionately high burden on health care resources because of the high risk of exacerbation, hospitalization, and death.^2^ There is increasing recognition that asthma is heterogeneous and comprises distinct phenotypes.^3-5^ Statistical techniques, such as factor and cluster analysis, have been used to dissect asthma heterogeneity and identify distinct clinical phenotypes.^4^ Although quantitative computed tomography (CT)–based disease phenotyping has been used in patients with chronic obstructive pulmonary disease,^6,7^ this has not yet been fully used in asthmatic patients. Quantitative CT techniques^8-10^ now enable assessment of the proximal airways,^9^ indirect assessment of the small airways,^11^ and assessment of the fractal geometry of the tracheobronchial tree.^12^

We hypothesized that asthma phenotypes, as determined by using quantitative CT measures of proximal airway remodeling and air trapping, have distinct clinical and physiologic features. Our study aims were (1) to compare quantitative CT measures of proximal airway remodeling and air trapping from volumetric paired inspiratory and expiratory CT scans between patients with severe asthma, patients with mild-to-moderate asthma, and healthy control subjects; (2) to compare the fractal dimension of segmented airway tree and terminal air space between patients with severe asthma, patients with mild-to-moderate asthma, and healthy control subjects; and (3) to use factor and cluster analysis with quantitative proximal and distal airway CT indices to generate novel asthma phenotypes and compare their clinical and physiologic features.

## Methods

Detailed methods are available in the Methods section in this article's Online Repository at www.jacionline.org.

### Subjects

Adults with asthma (severe asthma, n = 48; mild-to-moderate asthma, n = 17) and healthy control subjects (n = 30) were recruited into a single-center study. Asthma was confirmed by a respiratory physician based on history and supported by evidence of variable airflow obstruction, airway hyperresponsiveness, or both.^13^ Severe asthma was defined in accordance with American Thoracic Society (ATS) guidelines.^14^ Asthmatic patients who did not meet the ATS severe asthma definition were classified as having mild-to-moderate asthma. All patients with severe asthma (n = 48) had previously taken part in another study.^15^ Healthy subjects were asymptomatic and had no known respiratory illness, with normal spirometric results. All subjects underwent clinical characterization, including an extensive history, skin prick tests for common aeroallergens, peripheral blood tests, spirometry, full pulmonary function tests, methacholine challenge tests, and sputum induction.^16^ Asthma-related quality of life and asthma control were assessed by using the Asthma Quality of Life Questionnaire^17^ and Asthma Control Questionnaire.^18^ Informed consent was obtained from all subjects, and the study was approved by the Leicestershire, Northamptonshire, and Rutland Research Ethics Committee.

### CT imaging

**Fig E1:**
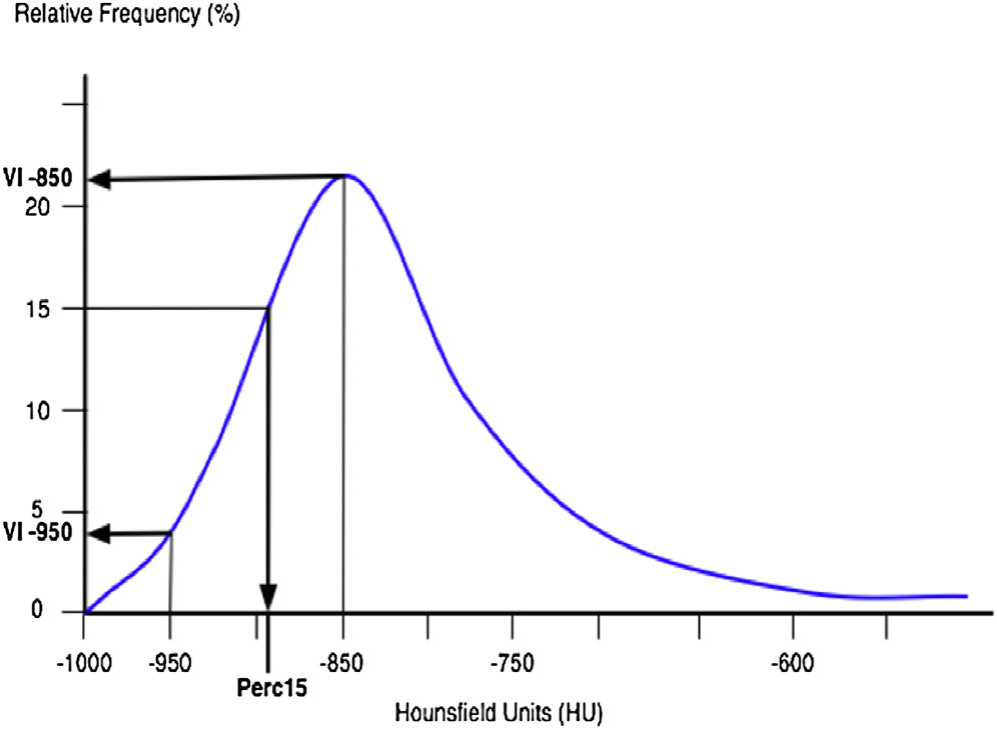
Densitometric indices. A cumulative voxel distribution histogram identifying derivation of various densitometric indices is shown. *Perc15*, 15th Percentile point.

**Fig E2:**
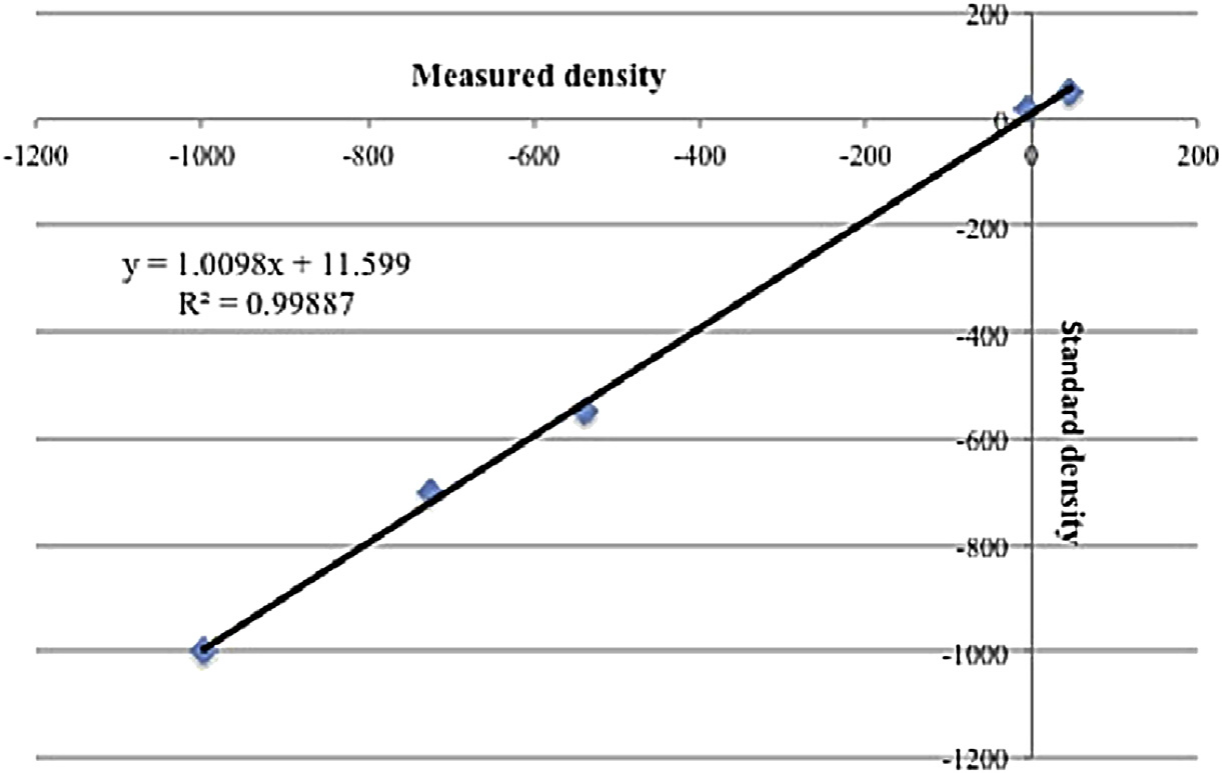
Standardization of densitometric indices. Regression equations were calculated from measurements of the densitometric standards (extrathoracic air, blood, and 3 electron density rods) for each CT scan of every subject in relation to the standard density measures of the densitometric standards. The regression equations derived were used to adjust all the densitometric indices for each CT scan of every subject. The figure illustrates derivation of regression equation for standardization of densitometric indices for one of the subjects.

**Fig E3:**
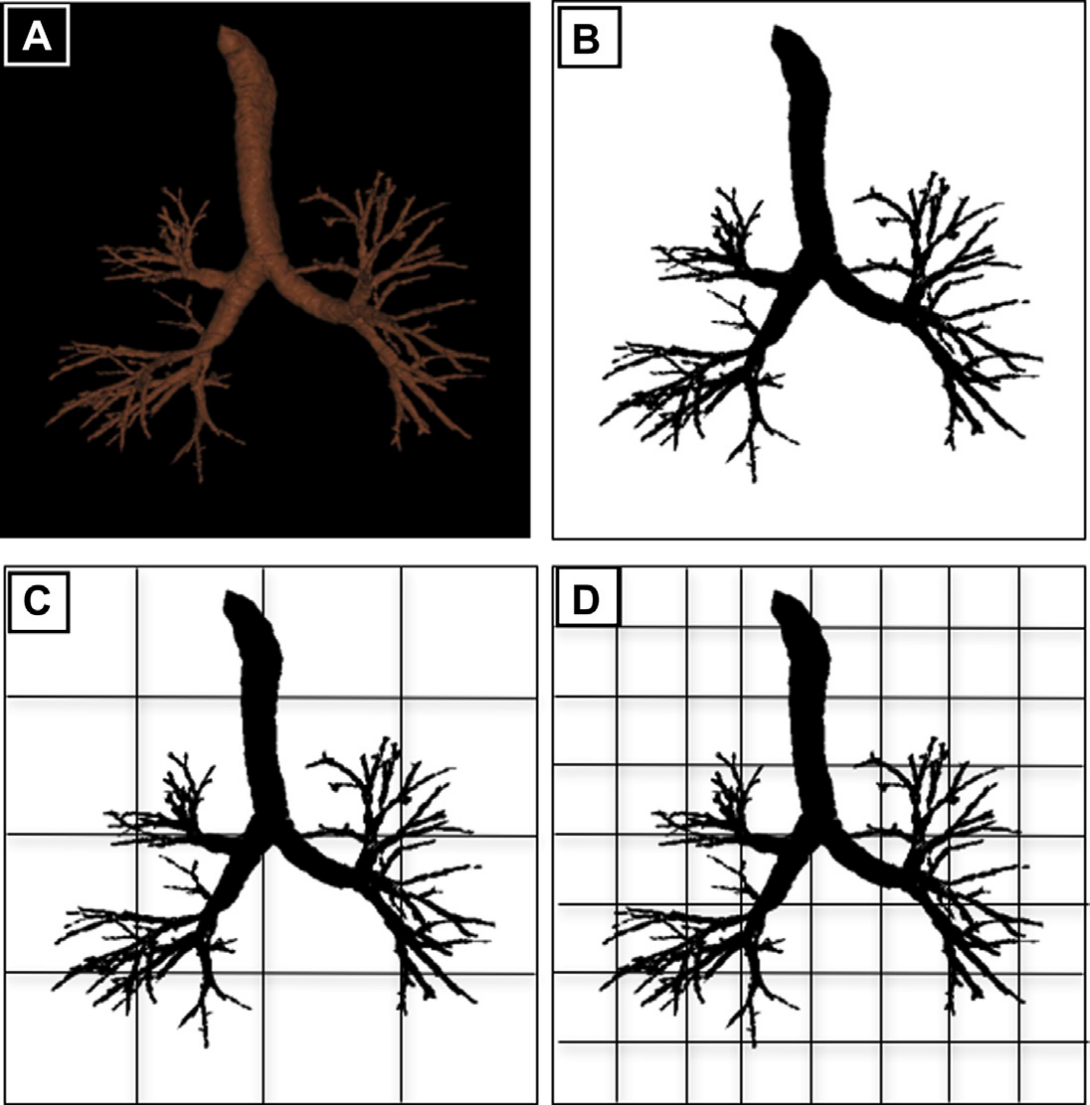
Fractal analysis of the segmented airway tree with FracLac (ImageJ) software. The JPEG image **(A)** of the segmented airway tree is binarized **(B)** by ImageJ software by assigning black pixel color for the airway tree and white pixel color for background. The box-counting algorithm places several grids of decreasing box size (**C** and **D**) over the ROI (ie, binarized image of the tracheobronchial tree to determine the fractal dimension).

**Fig E4:**
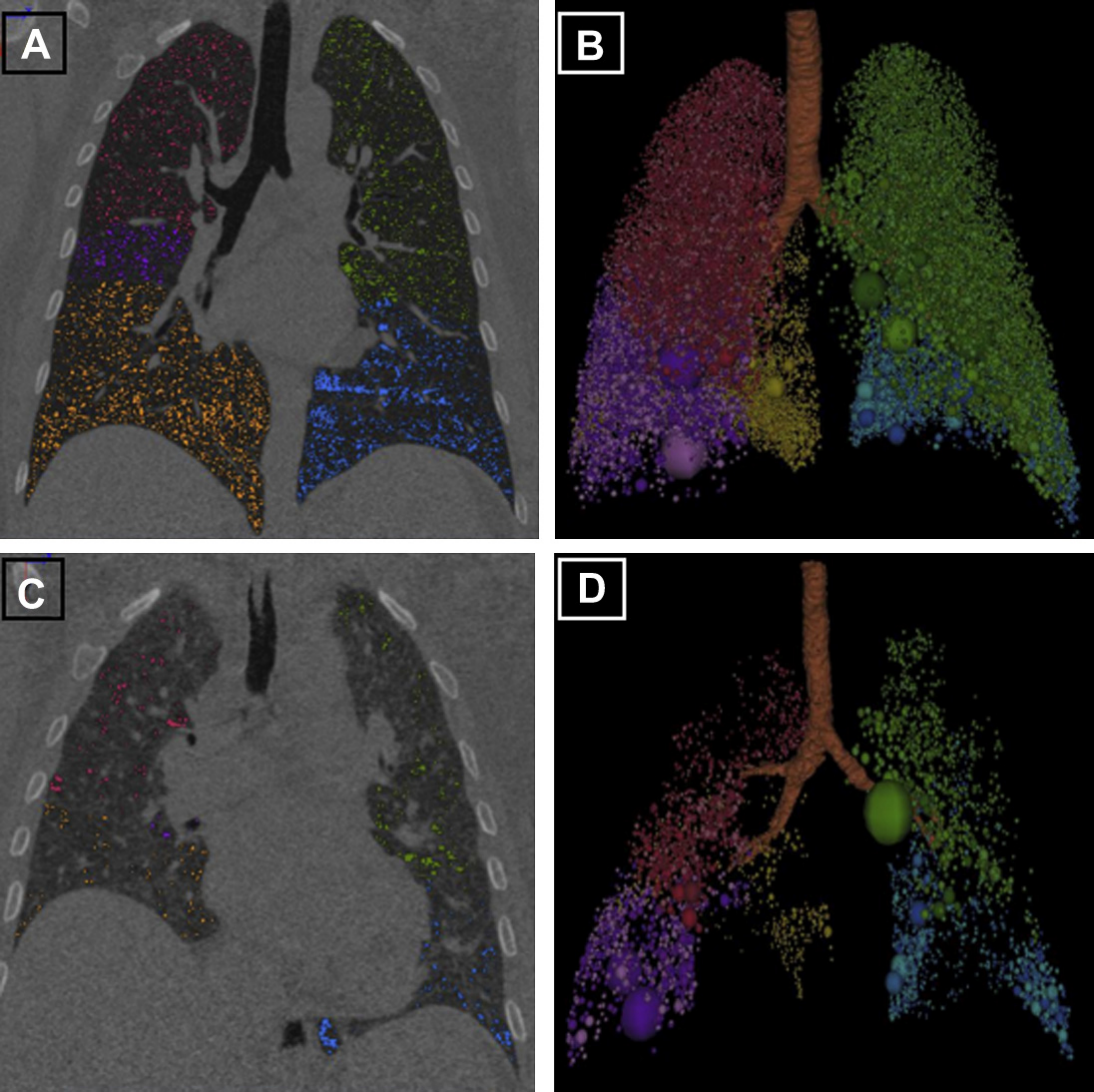
Fractal dimension of low attenuation areas in lungs. Coronal CT image showing areas of low attenuation less than the threshold CT attenuation value of −950 HU on inspiratory scans **(A)** and −850 HU on expiratory scans **(C)**, with each lobe coded with a different color. Respective low attenuation clusters are shown in **B** and **D**, which are used to assess the size and distribution of emphysematous lesions and air-trapping areas, respectively.

**Fig E5:**
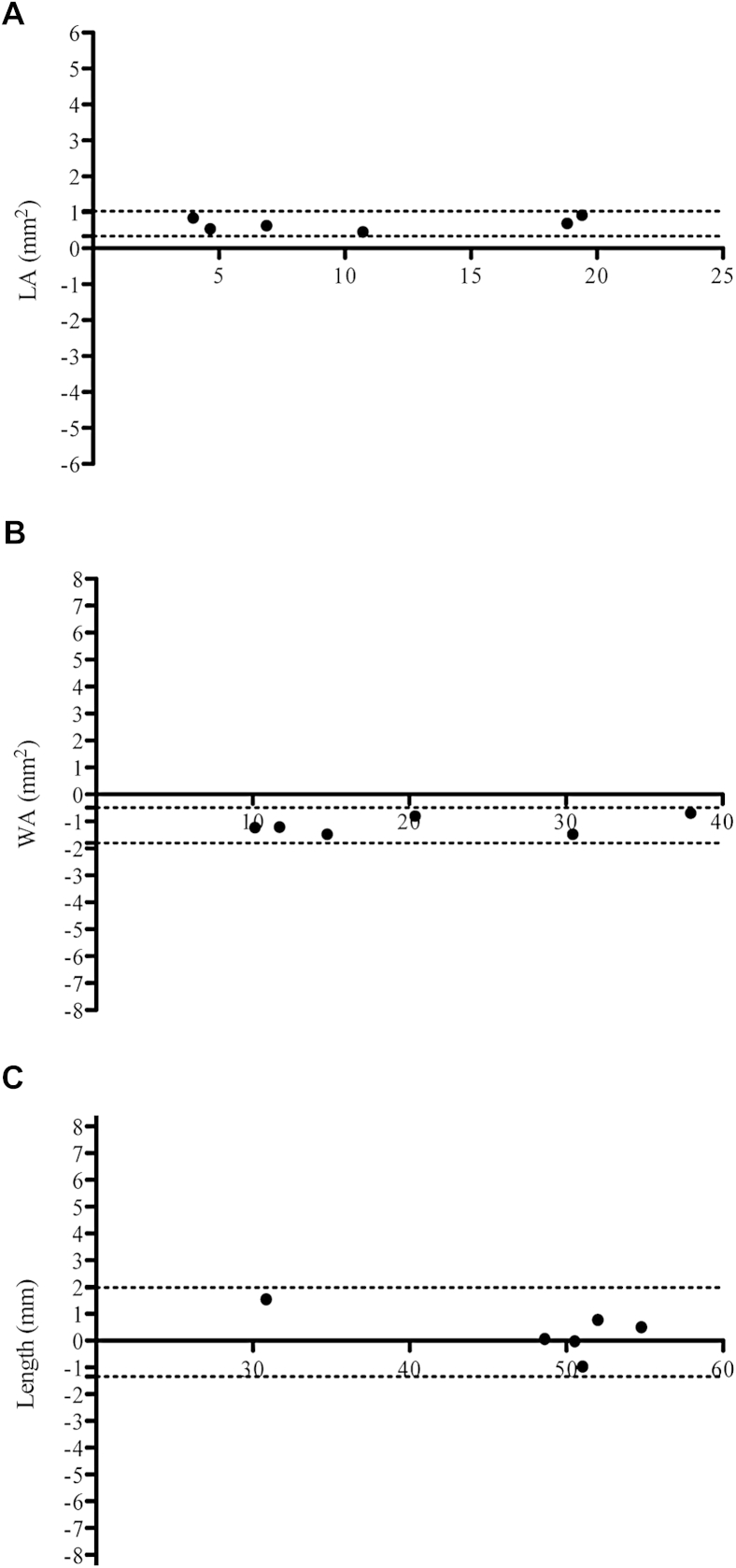
Bland-Altman plots and intraobserver repeatability with PW2 software. Differences between measures of LA **(A)**, WA **(B)**, and length **(C)** by single observers 2 months apart *(y-axis)* are plotted against the average of LA (Fig E5, *A*), WA (Fig E5, *B*), and length (Fig E5, *C*) measurements *(x-axis)*.

**Fig E6:**
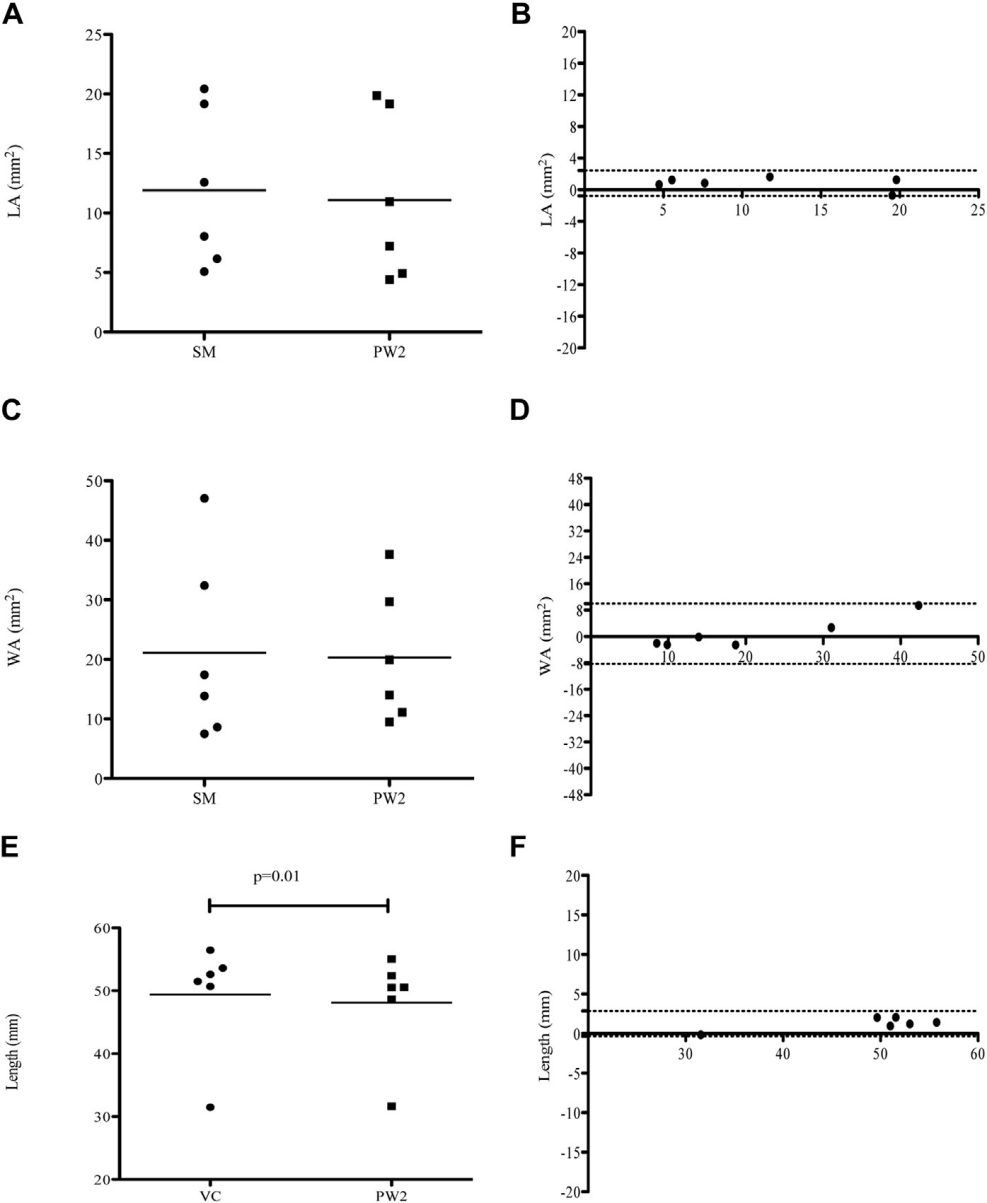
Accuracy of airway morphometry assessed by using PW2 software. Comparison of PW2 and stereomicroscopic measures of LA (**A** and **B**), WA (**C** and **D**), and length (**E** and **F**) by using the paired *t* test and Bland-Altman plots (difference *[y-axis]* vs the average *[x-axis]* measure using the 2 methods is plotted).

Volumetric whole-lung scans (Siemens Sensation 16; Siemens, Surrey, United Kingdom) were acquired at full inspiration and at the end of normal expiration. Details of CT acquisition and radiation safety (see Table E1 in this article's Online Repository at www.jacionline.org) are discussed in the Methods section in this article's Online Repository. Fully automated software, the Volumetric Information Display and Analysis (VIDA) Pulmonary Workstation, version 2.0 (PW2 software; VIDA Diagnostics, Coralville, Iowa; http://www.vidadiagnostics.com), was used for quantitative airway morphometry, lung densitometry (see Fig E1 in this article's Online Repository at www.jacionline.org) and calibrated by using density measures of air, blood, and electron density rods (see Fig E2 in this article's Online Repository at www.jacionline.org) and fractal dimension (see Figs E3 and E4 in this article's Online Repository at www.jacionline.org) analysis. The repeatability (see Fig E5 in this article's Online Repository at www.jacionline.org) and accuracy (see Fig E6 in this article's Online Repository at www.jacionline.org) of airway morphometry were assessed. Ninety-five percent CIs of mean lung density expiratory/inspiratory ratio (MLD E/I) among healthy control subjects was considered the normal range for CT air trapping. CT air trapping in asthmatic patients was graded based on MLD E/I values: (1) *severe*, greater than the upper limit of the 99.5% CI of the MLD E/I in healthy control subjects; (2) *moderate*, greater than the upper limit of the 98% CI of the MLD E/I in healthy control subjects; and (3) *mild*, greater than the upper limit of the 95% CI of the MLD E/I in healthy control subjects.

### Statistical analysis

Statistical analysis was performed with GraphPad Prism 5.00 (GraphPad Software, San Diego, Calif) and SPSS (SPSS, Chicago, Ill) software. Parametric data were expressed as means (SDs), and nonparametric data were described as medians (interquartile ranges). Log-transformed data are presented as geometric means (95% CIs). The χ^2^ and Fisher exact tests were used to compare ratios. One-way ANOVA with the Tukey correction (parametric data) and the Kruskal-Wallis test with the Dunn intergroup comparison (nonparametric data) were used to compare multiple groups. The Pearson correlation coefficient was used to determine airway structure and function relationships. Unsupervised multivariate modeling with principal component and cluster analysis was performed to extract factors that best describe the underlying relationship among the quantitative CT variables and determine cluster membership of all asthmatic patients. A 2-way random-effects model with absolute agreement intraclass correlation coefficients (ICCs) was used to assess single-measure reliability for the (1) lumen area (LA), wall area (WA), and length measurements of Leicester Airway Phantom tubes 4 to 9 by a single observer 2 months apart and (2) LA/WA and length measurements of Leicester Airway Phantom tubes 4 to 9 by using a steromicroscope and Vernier caliper, respectively, compared with PW2 software measurements. A *P* value of less than .05 was taken as statistically significant.

## Results

Baseline demographics and clinical characteristics of patients with severe (n = 48) or mild-to-moderate (n = 17) asthma and healthy control subjects (n = 30) are shown in Table I. Among the 3 groups, no significant differences were found in age, sex, body surface area (BSA), and smoking status.

### Proximal airway remodeling

**Fig E7:**
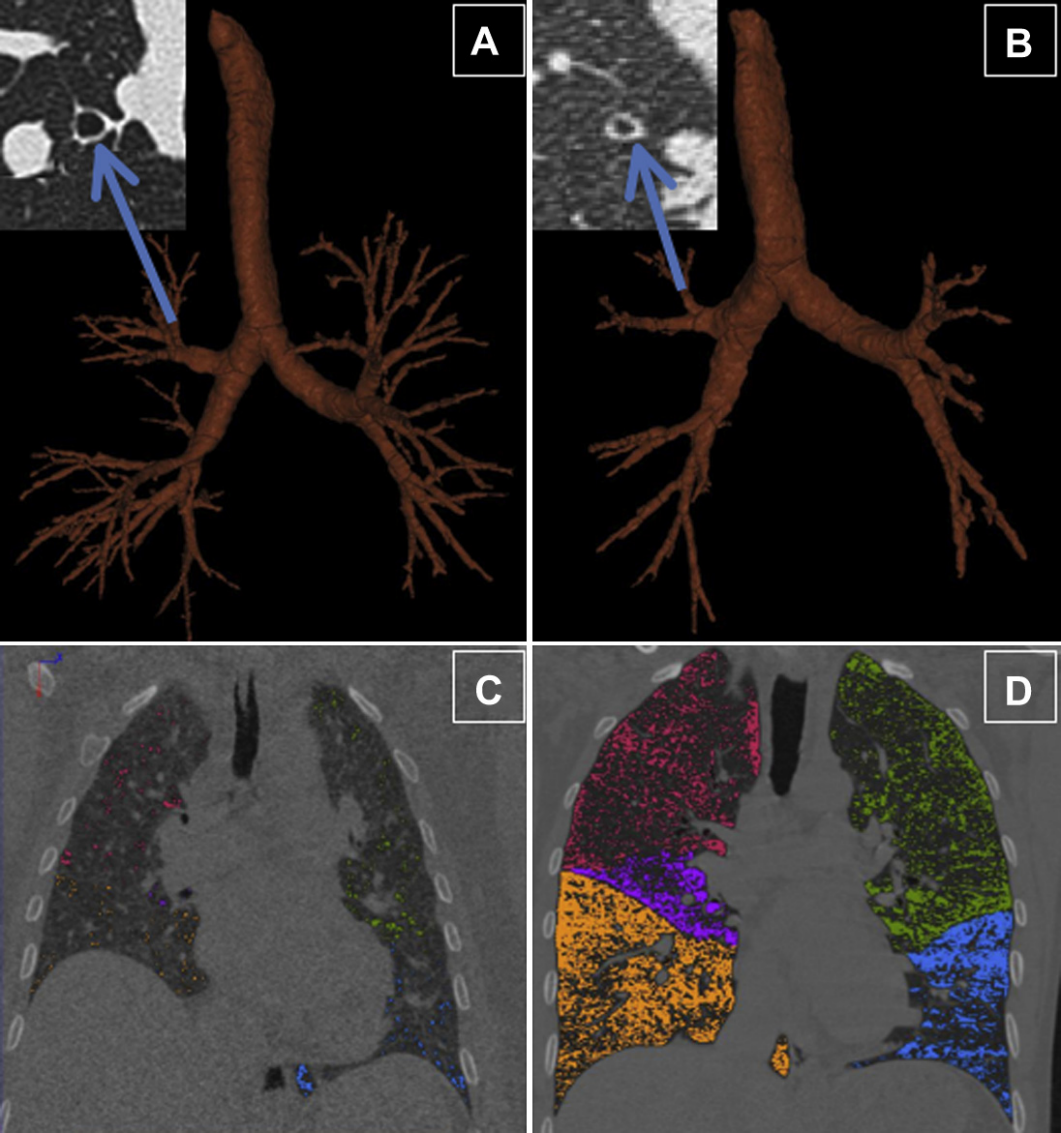
Quantitative CT in healthy subjects and patients with severe asthma. Three-dimensional reconstruction of an airway tree from a healthy subject **(A)** and a patient with severe asthma **(B)** using PW2 software, illustrating that in healthy subjects subsegmental airways can be reconstructed to more generations than in asthmatic patients. This is due to increased airway narrowing and closure in asthmatic patients. The *insets* illustrate the cross-section of RB1 in healthy subjects and patients with severe asthma, showing that the lumen is narrowed in asthmatic patients, with a decrease in total area and an increase in percentage WA but a relatively preserved WA. Coronal section of expiratory CT from a healthy subject **(C)** and a patient with severe asthma **(D)** showing the increased low attenuation areas (color coded according to lung lobes) in patients with severe asthma compared with healthy subjects.

On inspiratory CT scans, mean right upper lobe apical segmental bronchus (RB1) percentage wall volume (WV) was significantly higher in the groups with severe asthma and mild-to-moderate asthma compared with the healthy control subjects (62.4% [SD, 3.6%], 61.4% [SD, 2.9%], and 58.5% [SD, 3.6%], respectively; *P* < .0005). Patients with severe and mild-to-moderate asthma had smaller mean RB1 lumen volume (LV) in comparison with that seen in healthy control subjects (272.3 mm^3^ [SD, 112.6 mm^3^], 259.0 mm^3^ [SD, 53.3 mm^3^], and 366.4 mm^3^ [SD, 195.3 mm^3^], respectively; *P* = .007; see Fig E7 in this article's Online Repository at www.jacionline.org). No significant difference in RB1 dimension was found among the 2 asthma groups (Table II). Assessment of 3 other segmental bronchi (RB10, LB1+2, and LB10) on inspiratory CT scans revealed results similar to those for RB1. Mean (SD) LV or LA/BSA values of all the additional segmental bronchi assessed were significantly less in asthmatic patients compared with those seen in healthy control subjects (see Table E2 in this article's Online Repository at www.jacionline.org). RB10 and LB10 (but not LB1+2) percentage WV was significantly greater in asthmatic patients compared with that seen in healthy control subjects (see Table E2). RB1 lumen and wall dimensions showed good correlation with average lumen and wall dimensions of 4 (RB1, RB10, LB1+2, and LB10 [n = 44]) segmental bronchi, respectively (Pearson correlation coefficient, *r* = 0.7; *P* < .001). Hypothetical airway with an internal perimeter of 10 mm (Pi10) WA and hypothetical airway with an outer airway perimeter of 20 mm (Po20) percentage WA values were greater in patients with severe asthma compared with those seen in healthy control subjects (see Table E3 in this article's Online Repository at www.jacionline.org).

### Air trapping

CT-assessed lung volumes on inspiratory and expiratory scans were similar among the 3 groups (Table III). Air-trapping indices, MLD E/I values, and voxel index [VI] −850 changes on paired inspiratory and expiratory CT scans (VI_-850_ E-I) were significantly greater in asthmatic patients compared with those in healthy control subjects (Table III). The upper limits of the 99.5%, 98%, and 95% CIs of MLD E/I values in healthy control subjects were 0.862, 0.853, and 0.849, respectively. On assessment of all study subjects, there was no significant difference between expiratory CT lung volume and functional residual capacity (FRC) calculated on full lung function tests (mean, 2.9 L [SD, 0.9 L] vs 3.0 L [SD, 1.0 L]; *P* = .2). Inspiratory CT lung volume was less than total lung capacity (TLC), as assessed by using full lung function tests (mean, 5.2 L [SD, 1.4 L] vs 6.0 L [SD, 1.5 L]; *P* < .0005).

### Fractal dimension

**Fig E8:**
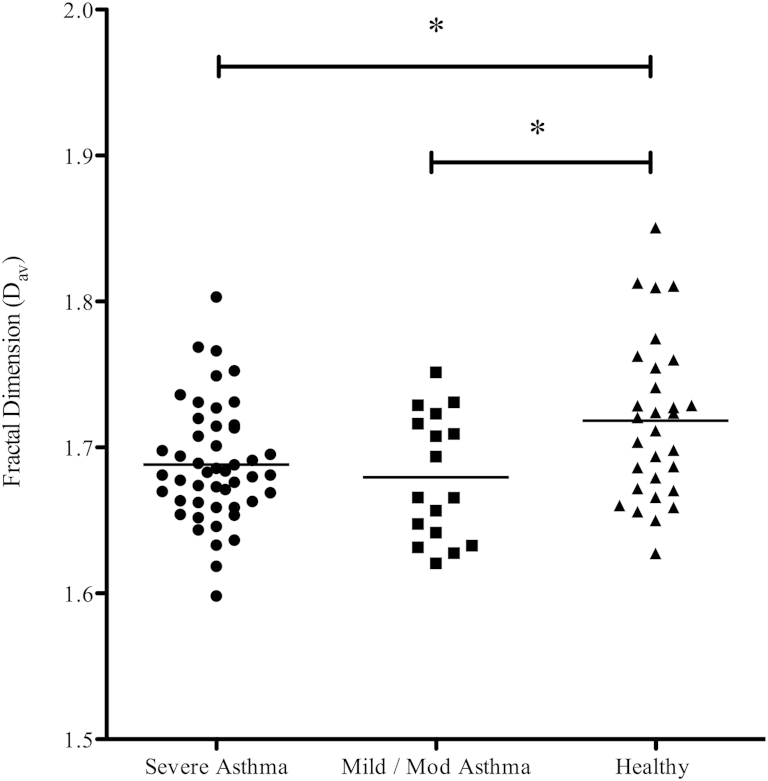
Fractal dimension of the segmented airway tree on an inspiratory CT scan in asthmatic patients and healthy subjects (∗*P* < .05, ANOVA).

On inspiratory CT, the average fractal dimension (D_av_) of the segmented airway tree was significantly less in asthmatic patients compared with that seen in control subjects, indicating decreased complexity of the branching airway tree in asthmatic patients (see Table E4 and Fig E8 in this article's Online Repository at www.jacionline.org). The fractal dimension of the low attenuation cluster at a threshold of −950 Hounsfield units (HU) on inspiratory scans and the fractal dimension of the low attenuation cluster at a threshold of −850 HU on expiratory scans were not different across the 3 groups (see Table E4).

### Univariate analysis to explore structure-function relationships

Good correlation was observed between air-trapping indices and the hypothetical airway measurements Pi10 WA or Po20 percentage WV (see Table E5 in this article's Online Repository at www.jacionline.org) but not between air-trapping indices and RB1 dimensions. Pi10 WA values inversely correlated with postbronchodilator FEV_1_/forced vital capacity ratios (*r* = −0.27, *P* < .05) and midexpiratory flow rates (*r* = −0.34, *P* < .05). A significant inverse correlation was also found between RB1 percentage WV and percentage residual volume (RV)/TLC (*r* = −0.35, *P* < .05, Table IV). MLD E/I values positively correlated with RV/TLC ratios (*r* = 0.46, *P* < .001) and negatively correlated with postbronchodilator FEV_1_ percent predicted values (*r* = −0.4, *P* < .001), postbronchodilator FEV_1_/forced vital capacity ratios (*r* = −0.48, *P* < .001), and midexpiratory flow rates (*r* = −0.6, *P* < .001). Other CT indices of air trapping also demonstrated similar correlation with lung function test results (Table IV). Disease duration, Asthma Control Questionnaire scores, and sputum eosinophil and neutrophil counts also showed correlations with CT air-trapping indices (Table IV).

### Unbiased CT phenotyping using factor and cluster analysis

**Fig 1:**
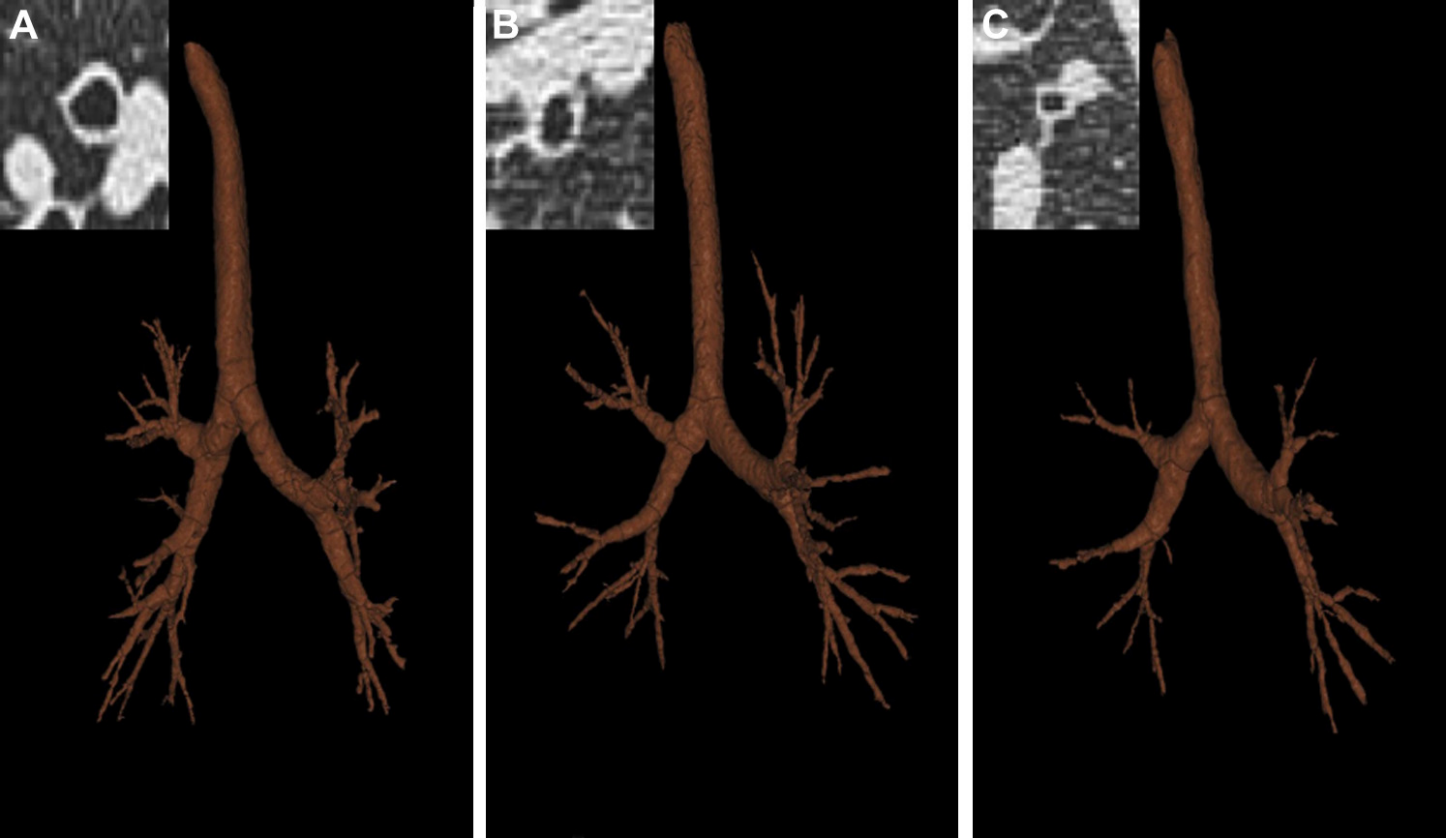
Proximal airway remodeling in asthma phenotypes. Pictures of segmented airway tree and RB1 CT cross-section *(insets)* of asthmatic patients from cluster 1 **(A)**, cluster 2 **(B)**, and cluster 3 **(C)** are shown.

The CT parameters were best described by 3 factors (see Table E6 in this article's Online Repository at www.jacionline.org). These factors were used in a cluster analysis, which identified 3 clusters. Clinical and quantitative CT characteristics of 3 asthma phenotypes determined by using cluster analysis are shown in Tables V and VI, respectively. All 3 asthma clusters demonstrate air trapping, suggesting the presence of small-airway disease. Clusters 1 and 3 demonstrate severe CT air trapping compared with the moderate CT air trapping seen in cluster 2. The normal range of expiratory VI −850 calculated from the 95% CI of the variable in healthy control subjects was 12.1% to 20.3%. The proportion of subjects with an expiratory VI −850 value of greater than 20.3% was higher in clusters 1 and 3 compared with that seen in cluster 2. Asthmatic patients in cluster 1, in addition to severe air trapping, had increased RB1 WV and LV but decreased RB1 percentage WV values. On the contrary, cluster 3 subjects, in addition to severe air trapping, had the smallest RB1 WV and LV values but highest RB1 percentage WV values in comparison with the other clusters (Fig 1). The fractal dimension of the segmented airway tree was highest in cluster 1. CT phenotyping performed by using average dimensions of 4 (RB1, RB10, LB1+2, and LB10; n = 44) segmental bronchi also identified 3 clusters with similar quantitative CT indices (cluster 1: LA/BSA of 14.3 mm^2^/m^2^ [1.5 mm^2^/m^2^] and WA/BSA of 21.0 mm^2^/m^2^ [1.3 mm^2^/m^2^]; cluster 2: LA/BSA of 13.1 mm^2^/m^2^ [0.7 mm^2^/m^2^] and WA/BSA of 19.6 mm^2^/m^2^ [0.8 mm^2^/m^2^]; and cluster 3: LA/BSA of 12.4 mm^2^/m^2^ [0.6 mm^2^/m^2^] and WA/BSA of 18.8 mm^2^/m^2^ [0.6 mm^2^/m^2^]).

No significant differences were found among the 3 clusters with regard to age, sex distribution, disease duration, smoking status, symptom score, severe exacerbation frequency, atopy, *Aspergillus* species sensitization, and sputum eosinophilic or neutrophilic inflammation. Subjects in clusters 1 and 3 were more often treated with long-acting β_2_-agonists compared with those in cluster 2. In clusters 1 and 3 the proportion of patients with severe asthma was greater. Subjects in clusters 1 and 3 had significantly higher RV/TLC percentages and lower prebronchodilator and postbronchodilator FEV_1_ percent predicted values compared with those in cluster 2. Subjects in cluster 3 had increased body mass index compared with the other groups. Bronchodilator response was significantly lower in cluster 2 subjects in comparison with those in other clusters. Clinical characteristics and quantitative CT indices of asthmatic patients in 3 asthma clusters were similar when analysis was performed after exclusion of subjects with a smoking history of greater than 10 pack years (see Table E7 in this article's Online Repository at www.jacionline.org).

## Discussion

We found that in asthmatic patients there was airway remodeling with reduced luminal volume and increased percentage WV compared with that seen in healthy subjects, irrespective of disease severity. This increase in percentage WV was largely driven by the reduction in luminal volume with no significant difference in WV between asthma and health, thus suggesting that airway remodeling reflects complex changes in the airway geometry rather than simply an increase in WV. Air trapping was increased in asthmatic patients compared with that seen in healthy subjects. The fractal dimension of the segmented airway tree was significantly less in asthmatic patients compared with that in healthy subjects on an inspiratory scan, indicating a loss of complexity and decrease in the space-filling ability of these airways. Using CT indices of proximal airway remodeling and air trapping, we identified 3 novel asthma phenotypes with distinct clinical and radiologic features.

Proximal airway remodeling, although an established feature of asthma,^9,19^ is heterogeneous, with variable changes in wall and lumen dimensions reported by several authors.^8-10,20^ Our results demonstrate lumen narrowing as a predominant feature of proximal airway remodeling in asthmatic patients, which is in keeping with our previous observations^10^ and those obtained by using optical coherence tomography.^21^ It is difficult to attribute the airway narrowing we observed in asthmatic patients to increased smooth muscle tone because all subjects were scanned after bronchodilator use. However, in some subjects airway smooth muscle tone might be partly refractory. We are confident that the changes observed in RB1 are generally reflective of changes throughout the airway tree as in spite of the heterogeneity of airway remodeling; RB1 has been shown to emulate changes in other proximal airways.^9,10,22^ Likewise, in our study the differences in RB1 dimensions between the asthmatic and healthy groups were reflected in airway remodeling patterns of 3 other proximal airways and 2 hypothetical airways, confirming that RB1 is a good surrogate for proximal airway remodeling.

Air-trapping indices were derived from paired inspiratory and expiratory scans after calibration by using density measures of air, blood, and electron density rods. X-ray tube aging and replacement might introduce errors in densitometric measures^23^ despite standard CT scanner quality assurance procedures.^24^ Therefore densitometric calibration is critical, and our study is the first to apply this for assessment of air trapping in asthmatic patients. The air trapping indices, MLD E/I and VI-850 E-I, were significantly greater in asthmatic patients compared with those seen in healthy subjects, which is in keeping with observations by other authors.^25,26^ In our study CT air trapping indices correlated with 2 hypothetical airways but not with any other proximal airway dimensions. Univariate analysis of CT air-trapping indices, in contrast to proximal airway remodeling, showed a much stronger correlation with lung function and disease duration. Previous studies^8-10,25,26^ have also demonstrated correlations between CT and clinical indices, thus highlighting the importance of structure-function relationships in asthmatic patients.

For the first time, we have used an unbiased method to determine 3 distinct asthma phenotypes based on CT measures of proximal airway remodeling and air trapping. Previously, we did not find any differences in proximal airway remodeling in 4 clinical severe asthma phenotypes,^10^ which prompted the current analysis. Both CT and physiologic measures demonstrate more severe air trapping in clusters 1 and 3 compared with cluster 2. Clusters 1 and 3 demonstrate poorer lung function compared with cluster 2. Moreover, lack of proximal airway remodeling in cluster 2 implies that this phenotype represents asthmatic patients with mild disease. Similarly, others report^9,27^ no significant differences in airway dimensions between the patients with mild-to-moderate asthma and healthy subjects. Wagner et al^28^ have shown a 7-fold increase in distal airway resistance in asthmatic patients with mild disease and normal spirometric results compared with healthy subjects. Taken together, these findings perhaps suggest that small-airway involvement in asthmatic patients precedes proximal airway remodeling. Cluster 1 subjects have significantly increased RB1 lumen and wall dimensions, whereas in contrast, cluster 3 subjects demonstrate luminal narrowing. Whether this represents pathologic dilatation of the airways in cluster 1 patients needs to be determined. The bronchiectasis phenotype of asthma has been described previously,^29-31^ which correlates negatively with FEV_1_^32^ and positively with air trapping.^33^ Similar to cluster 3 subjects, numerous CT studies have also demonstrated an increased percentage WA or percentage wall thickness in patients with severe asthma compared with healthy subjects.^8-10,22,25,34^ The phenotypes identified in our study do not align with clinical asthma phenotypes previously identified by our group^10^ and with traditional methods of asthma classification. The CT-derived phenotypes most likely represent a different aspect of asthma based on airway structural changes that are difficult to identify purely on the basis of demographic profiles, inflammatory indices, and lung function test results. Whether the CT-derived phenotypes we describe here represent distinct asthma endotypes^5^ with discrete pathogenic pathways or indicate a progressive disease captured at different stages of airway remodeling is uncertain and warrants further study.

Our finding of a decreased fractal dimension of the segmented airway tree in asthmatic patients compared with healthy subjects is consistent with similar analyses of the bronchial tree,^35^ peak expiratory flow time series,^36^ and fluctuation in daily fraction of exhaled nitric oxide levels^37^ in asthmatic patients. To determine the fractal dimension, we used the digital picture of the segmented airway tree obtained from CT scans using PW2 software, in contrast to the study by Boser et al,^35^ in which a digital picture of a silicone rubber cast of airways from postmortem subjects was used. Despite these differences in the method used by Boser et al and our group, the fractal dimension values obtained were similar. Reduced fractal dimension and hence the complexity of the airway tree might be fundamental in understanding the disordered physiology and exacerbation events^38^ associated with asthma. Fractal dimension of low attenuation clusters at a threshold of −950 HU on inspiratory scans can help detect early emphysema.^39^ In keeping with previous studies,^29,30,40^ there was no evidence of emphysema in asthmatic patients because both VI −950 values and fractal dimension of low attenuation clusters at a threshold of −950 HU were not significantly different compared with values seen in healthy subjects.

Our study has a number of potential limitations. Inspiratory and expiratory CT scans obtained were not spirometrically gated. We are confident that the differences observed in airway dimensions between asthmatic patients and healthy subjects in our study are not due to differences in lung volume because all subjects practiced breath holding before CT scans and no significant differences were found in inspiratory or expiratory lung volumes between groups. Expiratory CT–assessed lung volume was not significantly different from physiologically assessed FRC. Inspiratory CT–assessed lung volume, although lower than physiologically assessed TLC, was similar in healthy subjects and patients with mild-to-moderate or severe asthma. Studies have shown that it is unlikely that spirometric gating will further improve quantitative CT repeatability.^41^ Moreover, animal studies have demonstrated that airways do not distend isotropically with the lung, and after elimination of bronchial tone, airway lumen reaches a plateau at low transpulmonary pressure with trivial changes in LA on further increase of transpulmonary pressure.^42,43^ All 48 patients with severe asthma in our study had previously taken part in another study.^15^ This group fulfilled the ATS refractory asthma definition,^14^ had 2 or more severe exacerbations in the last year, and had evidence of eosinophilic airway inflammation in the last 2 years but not necessarily at the point of screening or randomization. These criteria were fulfilled by one third of the unselected severe asthma population assessed at our center. Therefore patients with severe asthma in our study might not be fully representative of the unselected population and would have a higher number of patients with frequent exacerbations and eosinophilic inflammation but are representative of at least a large proportion of the population with severe asthma. Moreover, unselected patients with mild-to-moderate asthma were also included in this study. Another potential criticism of our study is that the asthma clusters presented here were determined based on proximal airway dimensions of a single airway together with air-trapping indices and fractal dimensions. In keeping with previous studies,^9,10,22^ we found a close association between RB1 and other airways, and CT-derived clusters in the subset of subjects with available data using the average dimension of 4 segmental airways identified 3 clusters with similar quantitative CT indices to the clusters identified using RB1 dimensions alone. This suggests that despite heterogeneity, remodeling in RB1 reflects changes in other proximal airways. The number of healthy control subjects in this study is small, and therefore the normal ranges of quantitative CT indices generated might not be representative of the unselected healthy population. Larger data sets that include sufficient numbers of subjects to study the effects of age, sex, and ethnicity are required to generate population normal ranges.

Our study has further extended the tools to investigate asthma heterogeneity^44^ and for the first time used CT indices of proximal airway remodeling and air trapping to determine distinct asthma phenotypes. Consequently, our findings challenge the paradigm in asthma that airway wall remodeling is characterized by increased WV but rather suggest that in asthmatic patients there is an important component of air trapping coupled with distinct and polarized phenotypes of proximal airway dilatation or narrowing. Whether these changes occur in parallel to or as a consequence of small-airways disease need to be further investigated. Additionally, fractal analysis of the segmented airway tree and low attenuation clusters in asthmatic patients provide a global assessment of structural changes in airway and lung parenchyma. The inclusion of airway structure in asthma phenotyping might prove invaluable in patient stratification to inform underlying mechanisms of disease and for novel pharmacologic and nonpharmacologic treatments.

**Clinical implications**

Novel asthma phenotypes based on quantitative CT indices were identified. This might prove useful in patient selection for novel therapies.

## Figures and Tables

**Table E1:** Radiation risk associated with common radiologic examinations in the United Kingdom compared with research CT examinations

	DLP		ED		Equivalent period of natural background radiation‖	Lifetime additional risk of fatal cancer per examination¶
	75th Percentile	Mean	75th Percentile	Mean		
CXR (single PA radiograph)∗	x	x	x	0.02	3 d	1 in 1,000,000
Pelvic x-ray∗	x	x	x	0.7	4 mo	1 in 30,000
Barium swallow∗	x	x	x	1.5	8.5 mo	1 in 13,000
Head CT†	1015	820	2.1	1.7	9.7 mo	1 in 11,700
Abdomen CT†	399	312	6.0	4.7	2.1 y	1 in 10,000
Chest (cancer staging) CT†	536	479	7.6	6.8	3.1 y	1 in 6,700
Full thoracic CT (research)	x	93.6‡	x	1.5§	8.5 mo	1 in 13,000

*CTDI*_*vol*_, Volume CT dose index; *CXR*, chest x-ray; *DLP*, dose-length product; *ED*, effective dose; *PA*, posterior-anterior.

∗ Data are based on information from the Health Protection Agency, United Kingdom.^E3^

† Data are based on national survey of doses from CT scans in the United Kingdom in 2003.^E1^

‡ Calculated from scanner CTDI_vol_.

§ Calculated by using the ImPACT CT dosimetric calculator.^E2^

‖ Average natural background radiation in the United Kingdom is 2.2 mSv (range, 1.5-7.5 mSv).^E3^

¶ Approximate lifetime risk for patients 16 to 69 years old: pediatric patients multiply risks by approximately 2, and older patients divide risks by approximately 5.^E3^

**Table E2:** Other proximal airway dimensions

Inspiratory CT scans	Patients with severe asthma	Patients with mild-to-moderate asthma	Healthy control subjects	Significance (*P* value)
RB10	n = 45	n = 15	n = 30	
Wall area/BSA (mm^2^/m^2^)	17.6 (3.8)	18.2 (5.8)	19.2 (3.3)	.3
Lumen area/BSA (mm^2^/m^2^)	11.8 (3.7)	12.7 (4.5)	14.0 (3.8)	.05*
Total area/BSA (mm^2^/m^2^)	29.4 (7.4)	30.9 (10.2)	33.2 (6.9)	.1
Wall area (mm^2^)	33.8 (7.1)	34.4 (10.0)	37.1 (6.3)	.2
Lumen area (mm^2^)	22.4 (6.6)	24.0 (7.7)	27.1 (7.0)	.02*
Total area (mm^2^)	56.3 (13.3)	58.4 (17.4)	64.2 (12.9)	.06
Length (mm)	15.4 (7.2)	12.2 (4.8)	12.8 (4.8)	.1
Wall volume (mm^3^)	504.5 (218.3)	411.4 (171.4)	465.9 (172.7)	.3
LV (mm^3^)	332.5 (150.8)	284.5 (121.6)	334.7 (123.2)	.5
Total volume (mm^3^)	837.0 (365.4)	696.0 (289.1)	800.6 (290.4)	.4
% Wall volume	60.5 (3.1)	59.1 (2.8)	58.2 (3.4)	.008*
LB1+2	n = 43	n = 15	n = 26	
Wall area/BSA (mm^2^/m^2^)	21.9 (5.4)	20.5 (4.6)	20.1 (4.4)	.3
Lumen area/BSA (mm^2^/m^2^)	14.7 (6.3)	13.1 (3.6)	14.0 (4.2)	.6
Total area/BSA (mm^2^/m^2^)	36.6 (11.5)	33.6 (8.0)	34.1 (8.4)	.5
Wall area (mm^2^)	41.5 (10.4)	38.5 (7.8)	39.7 (9.3)	.5
Lumen area (mm^2^)	27.8 (12.5)	24.5 (6.0)	27.7 (8.7)	.6
Total area (mm^2^)	69.3 (22.5)	62.9 (13.5)	67.3 (17.8)	.6
Length (mm)	10.9 (3.3)	11.3 (4.2)	12.6 (4.2)	.2
Wall volume (mm^3^)	431.6 (110.1)	418.0 (138.6)	506.7 (242.4)	.1
LV (mm^3^)	280.7 (86.5)	265.0 (87.9)	359.6 (202.0)	.03*
Total volume (mm^3^)	712.3 (186.5)	683.0 (223.8)	866.3 (443.1)	.07
% Wall volume	60.9 (4.3)	61.3 (2.1)	59.5 (3.0)	.2
LB10	n = 42	n = 14	n = 27	
Wall area/BSA (mm^2^/m^2^)	19.2 (3.8)	19.6 (5.5)	20.6 (4.6)	.4
Lumen area/BSA (mm^2^/m^2^)	13.0 (4.2)	12.7 (4.8)	15.9 (5.5)	.03*
Total area/BSA (mm^2^/m^2^)	32.2 (7.7)	32.2 (10.1)	36.6 (9.9)	.1
Wall area (mm^2^)	36.0 (5.9)	37.3 (9.0)	40.0 (7.9)	.08
Lumen area (mm^2^)	24.4 (6.9)	24.1 (8.0)	30.7 (9.8)	.004*†
Total area (mm^2^)	60.4 (12.2)	61.4 (16.5)	70.8 (17.0)	.02*
Length (mm)	15.5 (6.7)	15.4 (4.5)	14.1 (5.2)	.6
Wall volume (mm^3^)	553.4 (243.1)	561.3 (192.7)	557.1 (213.0)	1.0
LV (mm^3^)	372.8 (181.3)	361.3 (143.0)	430.1 (209.4)	.4
Total volume (mm^3^)	926.2 (416.7)	922.6 (331.3)	987.2 (415.4)	.8
% Wall volume	60.2 (4.4)	61.2 (3.6)	57.2 (3.8)	.003*†

Data are expressed as means (SDs). *BSA*, Body surface area. *Intergroup comparisons* One-way ANOVA with the Tukey test to compare all pairs of columns: *P < .05, patients with severe asthma versus healthy control subjects; †*P* < .05, patients with mild-to-moderate asthma versus healthy control subjects; and ‡*P* < .05, patients with severe asthma versus patients with mild-to-moderate asthma.

**Table E3:** Dimensions of hypothetical airways (Pi10 and Po20)

	Patients with severe asthma (n = 48)	Patients with mild-to-moderate asthma (n = 17)	Healthy control subjects (n = 30)	Significance (*P* value)
Inspiratory				
Pi10 WA (mm^2^)	16.5 (1.7)	16.1 (1.5)	15.2 (1.3)	.002*
Po20 % WA	65.2 (2.3)	64.6 (2.4)	63.4 (1.6)	.002*

Data are expressed as means (SDs). *Intergroup comparisons* One-way ANOVA with the Tukey test to compare all pairs of columns: *P < .05, patients with severe asthma versus healthy control subjects; †*P* < .05, patients with mild-to-moderate asthma versus healthy control subjects; and ‡*P* < .05, patients with severe asthma versus patients with mild-to-moderate asthma.

**Table E4:** Fractal dimensions

	Patients with severe asthma (n = 48)	Patients with mild-to-moderate asthma (n = 17)	Healthy control subjects (n = 30)	Significance (*P* value)
Airway tree, inspiratory				
D_av_	1.688 (0.04)	1.680 (0.04)	1.718 (0.06)	.007*†
D_sc_	1.649 (0.03)	1.637 (0.03)	1.677 (0.05)	.001*†
D_e_	1.794 (0.05)	1.777 (0.04)	1.824 (0.07)	**.02**†
D_sce_	1.745 (0.04)	1.731 (0.03)	1.764 (0.05)	**.03**†
Terminal airspace				
Inspiratory LAC-D −950	1.972 (0.1)	1.956 (0.1)	1.987 (0.1)	.7
Expiratory LAC-D −850	1.812 (0.1)	1.802 (0.1)	1.813 (0.1)	.8

Data are expressed as means (SDs). *Intergroup comparisons* One-way ANOVA with the Tukey test to compare all pairs of columns: *P < .05, patients with severe asthma versus healthy control subjects; †*P* < .05, patients with mild-to-moderate asthma versus healthy control subjects; and ‡*P* < .05, patients with severe asthma versus patients with mild-to-moderate asthma. *D*_*av*_, Fractal dimension, which is averaged over all 10 global scan locations; *D*_*e*_, same as D_av_ but for each grid size, the box count that required the lowest number of boxes was used; *D*_*sc*_, same as D_av_ but corrected for periods of no change for the log-log plot of box size and count; *D*_*sce*_, combination of all the above; *LAC-D −850*, Fractal dimension of low attenuation cluster at a threshold of −850 HU; *LAC-D −950*, fractal dimension of low attenuation cluster at a threshold of −950 HU.

**Table E5:** Univariate analysis of the relationship between proximal airway dimensions on inspiratory scans and CT air-trapping indices (n = 65)

	Expiratory mean lung density (HU)	Expiratory VI −850 (%)	MLD E/I	VI_−850/−950_ E-I (%)	VI_−850_ E-I (%)
RB1 LA/BSA (mm^2^/m^2^)	−0.13	0.07	0.1	−0.07	−0.01
RB1 WA/BSA (mm^2^/m^2^)	−0.07	−0.02	0.11	−0.09	−0.01
RB1 TA/BSA (mm^2^/m^2^)	−0.1	0.02	0.11	−0.09	−0.01
RB1 LV (mm^3^)	−0.15	0.16	0.03	−0.04	−0.04
RB1 wall volume (mm^3^)	−0.01	0.14	0.04	−0.04	−0.04
RB1 total volume (mm^3^)	−0.14	0.15	0.04	−0.04	−0.04
RB1 % wall volume	0.14	−0.2	0.002	0.04	0.03
Pi10 wall area (mm^2^)	−0.16	0.29*	0.28*	0.40†	0.35†
Po20 % wall area	−0.05	0.17	0.28*	0.47†	0.42†

Data are expressed as the Pearson correlation coefficient: *P < .05 and †*P* < .001. *TA*, Total area.

**Table E6:** Component loading of selected variables

	Components		
	1	2	3
RB1 LA/BSA	0.949*	0.020	0.117
RB1 LV	0.935	0.021	−0.024
RB1 TA	0.905	0.050	0.231
RB1 WA/BSA	0.876	0.021	0.249
RB1 WV	0.831	0.045	0.049
RB1 % WV	−0.682	0.035	0.180
MLD E/I	0.054	0.937*	−0.101
VI_-850/−950_ E-I	−0.102	0.901	0.090
Expiratory VI −850	0.138	0.757	−0.427
Expiratory LAC-D −850	−0.063	−0.301	0.745*
Inspiratory D_av_	0.244	0.054	0.610

*Rotated component matrix* Extraction method: principal component analysis Rotation method: Varimax with Kaiser normalization Component loading of the 11 original variables with the 3 main components derived by means of factor analysis in the 62 asthmatic patients is shown, with the predominant variable in each component indicated by an *asterisk*. *LAC-D −850*, Fractal dimension of low attenuation cluster at a threshold of −850 HU; *TA*, total area.

**Table E7:** Asthma phenotypes (subjects with a smoking history of >10 pack years were excluded)

	Asthma cluster 1: Severe air trapping, bronchial wall thickening, and bronchial lumen dilatation (n = 10)	Asthma cluster 2: Moderate air trapping (n = 33)	Asthma cluster 3: Severe air trapping and bronchial lumen narrowing (n = 15)	Significance (*P* value)
Age (y)	52.1 (10.1)	49.2 (13.3)	54.3 (8.9)	.4
Sex (M/F)	7/3	17/16	6/9	.8
Postbronchodilator FEV_1_ (% predicted)	61.4 (17.0)	84.2 (20.2)	72.2 (16.3)	.005*
Postbronchodilator FEV_1_/FVC ratio (%)	65.6 (13.3)	72.8 (10.6)	68.4 (9.5)	.2
Midexpiratory flow (L/s)	1.7 (1.2)	2.1 (0.9)	1.9 (0.9)	.6
RV (L)	2.9 (0.8)	1.7 (0.7)	2.0 (0.9)	.006*
TLC (L)	6.4 (1.4)	5.8 (1.4)	5.7 (2.1)	.6
RV/TLC (%)	45.2 (11.0)	29.2 (6.5)	35.4 (9.6)	<.005*
RB1 % wall volume	57.9 (2.9)	62.0 (2.6)	64.9 (2.9)	<.005*†‡
RB1 wall volume (mm^3^)	544.7 (96.8)	455.7 (121.3)	331.3 (107.3)	**<.005**†‡
RB1 wall volume (% greater than upper 95% CI of healthy control subjects)	30	15	7	.3
RB1 LV (mm^3^)	397.0 (81.1)	280.1 (78.2)	179.6 (56.7)	<.005*†‡
RB1 LV (% less than lower 95% CI of healthy control subjects)	10	64	93	**<.005**
Expiratory VI −850 (%)	24.9 (16.4)	17.5 (11.9)	23.4 (11.5)	.2
Expiratory VI −850 (% greater than upper 95% CI of healthy control subjects)	70	30	60	**.03**
MLD E/I	0.881 (0.06)	0.858 (0.06)	0.861 (0.05)	.5
MLD E/I (% greater than upper 95% CI of healthy control subjects)	80	55	67	.3

Data are expressed as means (SDs). Pearson χ^2^ and Fisher exact tests were used to compare ratios. *FVC*, Forced vital capacity. *Intergroup comparisons* One-way ANOVA with the Tukey test to compare all pairs of columns: *P < .05, asthma cluster 1 versus asthma cluster 2; †*P* < .05, asthma cluster 1 versus asthma cluster 3; and ‡*P* < .05, asthma cluster 2 versus asthma cluster 3.

**Table I:** Clinical characteristics of asthmatic patients and healthy subjects

	Patients with severe asthma (n = 48)	Patients with mild-to-moderate asthma (n = 17)	Healthy control subjects (n = 30)	Significance (*P* value)
Age (y)	50.7 (9.9)	53.0 (16.3)	56.9 (12.5)	.09
Sex (M/F)	24/24	10/7	16/14	.8
BMI (kg/m^2^)	28.8 (6.4)	28.3 (5.2)	28.3 (5.2)	.9
BSA (m^2^)	1.9 (0.2)	1.9 (0.2)	1.9 (0.2)	1.0
Disease duration (y)	26.8 (16.3)	33.2 (5.7)	x	.2
Smoking status (%)				
Never	77	59	52	.2
Exsmoker	21	35	41	
Current smoker	2	6	7	
Smoking history >10 pack years (%)	6	12	13	.3
Atopy (%)	67	82	18	**<.0005**
*Aspergillus* species sensitization (%)	23	36	7	.08
Severe exacerbations/y†	3.0 (1.0-5.3)	1.0 (0-2.5)	x	**.007**
Modified ACQ score (symptoms only)	2.6 (1.3)	1.8 (1.4)	x	**.04**
AQLQ score	8.3 (25.1)	5.2 (1.4)	x	.6
Prebronchodilator FEV_1_ (% predicted)	69.2 (20.1)	79.0 (23.4)	110.9 (15.9)	**<.0005**‡§
Prebronchodilator FEV_1_/FVC ratio (%)	67.9 (12.1)	69.7 (10.3)	78.3 (5.6)	**<.0005**‡§
Postbronchodilator FEV_1_ (% predicted)	74.4 (19.2)	81.3 (22.7)	112.7 (17.3)	**<.0005**‡§
Postbronchodilator FEV_1_/FVC ratio (%)	69.9 (11.9)	69.8 (10.4)	79.9 (6.0)	**<.0005**‡§
Bronchodilator response (%)	9.6 (14.7)	3.5 (6.8)	1.6 (3.5)	**.0007**‡
Midexpiratory flow (L/s)	2.0 (1.1)	1.8 (0.9)	3.2 (0.9)	**<.0005**‡§
Vital capacity (L)	3.7 (1.1)	4.0 (0.8)	4.2 (0.9)	.2
Functional residual capacity (L)	3.0 (1.2)	2.9 (0.9)	3.1 (0.9)	.9
RV (L)	2.1 (0.9)	2.0 (0.9)	2.2 (0.8)	.8
TLC (L)	5.8 (1.7)	5.9 (1.4)	6.3 (1.2)	.6
RV/TLC (%)	35.7 (12.3)	31.2 (9.4)	33.6 (15.3)	.7
Methacholine PC_20_ (mg/mL)∗	2.0 (0.7-6.0)	2.4 (0.8-7.3)	24.4 (17.6-33.7)	**<.0005**‡§
Feno (ppb)∗	36.1 (27.7-47.0)	26.4 (18.6-37.5)	27.2 (21.1-34.7)	.2
Total IgE (kU/L)∗	179.0 (128.7-248.9)	137.0 (68.5-273.8)	25.5 (15.8-41.0)	**<.0005**‡§
Inhaled CS (%)	100	82	x	**.02**
Inhaled CS dose, BDP (μg/24 h)†	2000 (1600-2000)	1000 (600-2000)	x	**.004**
LABA (%)	94	71	x	**.03**
Oral CS (%)	68	0	x	**<.0005**
Montelukast (%)	32	0	x	**.007**
Theophylline (%)	40	24	x	.3
Sputum eosinophils (%)∗	4.3 (2.5-7.5)	2.0 (0.8-4.8)	0.7 (0.4-.1)	**<.0005**‡
Sputum total neutrophils × 10^6^ (cells/g)	2.8 (7.0)	4.0 (5.2)	2.9 (3.9)	.9

Data are expressed as means (SDs). The Pearson χ^2^ and Fisher exact test were used to compare ratios. Beclomethasone dipropionate equivalents are as follows: fluticasone, 2:1; budesonide, 1.25:1; mometasone, 1.25:1; QVAR, 2:1; and ciclesonide, 2.5:1. *ACS*, Asthma Control Score; *BDP*, beclomethasone dipropionate; *BMI*, body mass index; *CS*, corticosteroid; *Feno*, fraction of exhaled nitric oxide; *FVC*, forced vital capacity; *LABA*, long-acting β_2_-agonist.

∗ Geometric mean (95% CI).

† Median (interquartile range). *Intergroup comparisons* For parametric data, 1-way ANOVA with the Tukey test to compare all pairs of columns: ‡*P* < .05, patients with severe asthma versus healthy control subjects; §*P* < .05, patients with mild-to-moderate asthma versus healthy control subjects; and ‖*P* < .05, patients with severe asthma versus patients with mild-to-moderate asthma. For nonparametric data, Kruskal-Wallis test with the Dunn multiple comparison test to compare all pairs of columns: ‡*P* < .05, patients with severe asthma versus healthy control subjects; §*P* < .05, patients with mild-to-moderate asthma versus healthy control subjects; and ‖*P* < .05, patients with severe asthma versus patients with mild-to-moderate asthma, Mann-Whitney *U* test.

**Table II:** RB1 dimensions of asthmatic patients and healthy subjects

	Patients with severe asthma (n = 48)	Patients with mild-to-moderate asthma (n = 17)	Healthy control subjects (n = 30)	Significance (*P* value)
Inspiratory				
Wall area/BSA (mm^2^/m^2^)	18.2 (5.5)	18.5 (3.7)	18.7 (5.2)	.9
Lumen area/BSA (mm^2^/m^2^)	11.3 (4.4)	11.7 (3.0)	13.7 (5.2)	.08
Total area/BSA (mm^2^/m^2^)	29.5 (9.7)	30.2 (6.4)	32.3 (10.3)	.4
Wall area (mm^2^)	34.8 (10.6)	35.6 (8.0)	36.1 (10.7)	.8
Lumen area (mm^2^)	21.6 (8.4)	22.6 (6.5)	26 (10.3)	.06
Total area (mm^2^)	56.4 (18.7)	58.2 (14.2)	62.6 (20.8)	.4
Length (mm)	12.8 (2.7)	11.8 (2.4)	13.7 (4.4)	.2
Wall volume (mm^3^)	437.6 (144.0)	412.9 (88.5)	496.3 (222.1)	.2
LV (mm^3^)	272.3 (112.6)	259.0 (53.3)	366.4 (195.3)	.007*†
Total volume (mm^3^)	709.9 (252.4)	672.0 (134.9)	862.8 (414.6)	**.05**
Wall volume (%)	62.4 (3.6)	61.4 (2.9)	58.5 (3.6)	<.0005*†

Data are expressed as means (SDs). *BSA*, Body surface area. *Intergroup comparisons* One-way ANOVA with the Tukey test to compare all pairs of columns: *P < .05, patients with severe asthma versus healthy control subjects; †*P* < .05, patients with mild-to-moderate asthma versus healthy control subjects; and ‡*P* < .05, patients with severe asthma versus patients with mild-to-moderate asthma.

**Table III:** Densitometric indices in asthmatic patients and healthy subjects

	Patients with severe asthma (n = 48)	Patients with mild-to-moderate asthma (n = 17)	Healthy control subjects (n = 30)	Significance (*P* value)
Inspiratory				
Lung volume (L)	5.15 (1.5)	5.02 (1.4)	5.49 (1.2)	.4
Mean lung density (HU)	−830.9 (40.8)	−831.5 (42.7)	−837.8 (26.2)	.7
VI −950 (%)	11.2 (5.8)	11.9 (7.6)	10.1 (5.5)	.6
Percentile 15 (HU)	−932.8 (30.4)	−934.3 (28.2)	−930.1 (22.3)	.9
VI −850 (%)	59.1 (15.5)	59.9 (17.2)	61.8 (13.1)	.8
Expiratory				
Lung volume (L)	2.9 (1.0)	2.9 (1.1)	2.8 (0.8)	.9
Mean lung density (HU)	−713.3 (53.2)	−719.5 (63.0)	−695.6 (55.0)	.3
Air-trapping indices				
Expiratory VI −850 (%)	19.7 (11.5)	22.0 (18.0)	16.2 (11.5)	.3
MLD E/I	0.861 (0.05)	0.866 (0.07)	0.830 (0.06)	**.04**
VI_−850/−950_ E-I (%)	−30.4 (9.7)	−29.1 (18.8)	−36.9 (10.2)	**.04**
VI_−850_ E-I (%)	−39.1 (11.5)	−37.8 (20.1)	−45.6 (12.0)	.08

Data are expressed as means (SDs). *Intergroup comparisons* One-way ANOVA with the Tukey test was used to compare all pairs of columns. All densitometric indices were standardized for extrathoracic air, blood, and 3 electron density rods.

**Table IV:** Univariate analysis of the relationship between clinical indices and proximal airway dimensions on inspiratory scans or CT air trapping (n = 65)

	Post-BD FEV_1_ (% predicted)	Post-BD FEV_1_/FVC ratio (%)	Midexpiratory flow (L/s)	RV/TLC (%)	Disease duration (y)	Sputum total neutrophils × 10^6^ (cells/g)	Log sputum eosinophil count	ACQ
Proximal airway dimensions (inspiratory scan)								
RB1 LA/BSA (mm^2^/m^2^)	−0.10	−0.06	−0.02	0.30	−0.02	0.13	0.08	−0.17
RB1 WA/BSA (mm^2^/m^2^)	−0.07	−0.04	−0.05	0.20	0.08	0.10	0.08	−0.13
RB1 TA/BSA (mm^2^/m^2^)	−0.09	−0.06	−0.04	0.25	0.03	0.11	0.08	−0.15
RB1 LV (mm^3^)	−0.01	−0.06	0.08	0.21	−0.04	0.10	−0.03	−0.18
RB1 wall volume (mm^3^)	0.05	−0.05	0.08	0.11	0.05	0.06	−0.04	−0.16
RB1 total volume (mm^3^)	0.03	−0.06	0.08	0.15	0.01	0.08	−0.04	−0.17
RB1 % WV	0.08	0.05	−0.02	−0.35*	0.19	−0.11	−0.06	0.19
Pi10 WA (mm^2^)	−0.21	−0.27*	−0.34*	0.21	−0.07	0.04	−0.10	0.17
Po20 % WA	−0.11	−0.10	−0.14	0.02	0.05	0.03	−0.22	0.19
Air-trapping indices								
Expiratory mean lung density (HU)	0.45†	0.64†	0.47†	−0.63†	−0.35*	0.03	−0.38*	−0.10
Expiratory VI −850 (%)	−0.48†	−0.68†	−0.48†	0.64†	0.27	0.10	0.23	0.15
MLD E/I	−0.40†	−0.48†	−0.60†	0.46†	0.29*	0.17	0.02	0.23
VI_−850/−950_ E-I (%)	−0.30*	−0.32*	−0.41†	0.34*	0.03	0.34*	−0.23	0.30*
VI_−850_ E-I (%)	−0.22	−0.15	−0.33*	0.25	−0.03	0.37*	−0.27	0.28*

Data are expressed as the Pearson correlation coefficient: *P < .05 and †*P* < .001. *ACQ*, Asthma Control Questionnaire; *BD*, bronchodilator; *FVC*, forced vital capacity; *TA*, total area.

**Table V:** Clinical characteristics of asthma phenotypes

	Asthma cluster 1: Severe air trapping, bronchial wall thickening, and bronchial lumen dilatation (n = 11)	Asthma cluster 2: Moderate air trapping (n = 34)	Asthma cluster 3: Severe air trapping and bronchial lumen narrowing (n = 17)	Significance (*P* value)
Age (y)	51.9 (9.6)	49.3 (13.1)	54.7 (10.4)	.3
Sex (M/F)	4:7	17:17	9:8	.7
BMI (kg/m^2^)	25.0 (3.4)	28.3 (5.5)	31.5 (7.3)	**.02**§
BSA (m^2^)	1.9 (0.2)	1.9 (0.2)	2.0 (0.3)	.3
Patients with severe asthma (%)	81.8	58.8	94.1	**.02**
Disease duration (y)	26.3 (16.1)	26.4 (16.1)	27.2 (17.5)	1.0
Smoking status (%)				
Never	60	74	82	.6
Exsmoker	40	23	18	
Current smoker	0	3	0	
Smoking history >10 pack years (%)	8	3	13	.5
Atopy (%)	73	69	69	1.0
*Aspergillus* species sensitization (%)	36	17	29	.4
Severe exacerbations/y†	2.5 (1-4.25)	1.5 (0-5.0)	2 (1.0-6.5)	.6
Modified ACQ score (symptoms only)	2.1 (0.9)	2.0 (1.5)	2.8 (1.3)	.1
AQLQ score	4.9 (1.0)	5.0 (1.4)	4.1 (1.1)	.09
Prebronchodilator FEV_1_ (% predicted)	58.0 (17.3)	80.7 (19.9)	64.0 (19.6)	**.001**‡‖
Prebronchodilator FEV_1_/FVC ratio (%)	64.8 (12.9)	71.1 (9.7)	67.0 (13.5)	.2
Postbronchodilator FEV_1_ (% predicted)	63.8 (17.7)	83.8 (20.0)	70.6 (16.6)	**.005**‡‖
Postbronchodilator FEV_1_/FVC ratio (%)	67.0 (13.2)	72.8 (10.4)	67.2 (10.7)	.2
Bronchodilator response (%)	11.1 (11.8)	4.4 (7.2)	13.9 (20.8)	**.04**‖
Midexpiratory flow (L/s)	2.0 (1.4)	2.1 (0.9)	1.9 (1.0)	.8
Vital capacity (L)	3.5 (1.0)	4.0 (1.0)	3.6 (1.1)	.4
Functional residual capacity (L)	3.6 (1.2)	2.7 (0.8)	2.7 (1.1)	.06
RV (L)	2.7 (0.9)	1.7 (0.7)	2.1 (1.0)	**.02**‡
TLC (L)	6.2 (1.4)	5.7 (1.4)	5.8 (1.9)	.8
RV/TLC (%)	43.5 (11.2)	29.1 (6.3)	35.5 (10.6)	**.001**‡
Methacholine PC_20_ (mg/mL)∗	1.5 (0.1-16.4)	3.7 (1.2-11.5)	0.7 (0.07-5.6)	.2
Feno (ppb)∗	37.5 (20.0-70.2)	30.5 (22.6-41.3)	38.8 (24.5-61.4)	.6
Total IgE (kU/L)∗	227.2 (81.3-634.9)	139.2 (97.3-199.1)	217.9 (110.0-432.4)	.3
Inhaled CS (%)	100	91	100	.3
Inhaled CS dose BDP (μg/24 h)†	2000 (1450-2000)	2000 (1000-2000)	2000 (1500-2000)	.2
LABA (%)	100	77	100	**.03**
Oral CS (%)	60	41	53	.5
Montelukast (%)	10	24	29	.5
Theophylline (%)	40	29	35	.8
Sputum eosinophils (%)∗	2.8 (0.9-8.4)	3.3 (1.6-6.7)	5.7 (1.5-20.6)	.6
Sputum total neutrophils × 10^6^ (cells/g)	2.0 (1.8)	2.4 (3.6)	2.4 (2.1)	.9

Data are expressed as means (SDs). Pearson χ^2^ and Fisher exact tests were used to compare ratios. Beclomethasone dipropionate equivalents are as follows: fluticasone, 2:1; budesonide, 1.25:1; mometasone, 1.25:1; QVAR, 2:1; and ciclesonide, 2.5:1. *ACQ*, Asthma Control Questionnaire; *AQLQ*, Asthma Quality of Life Questionnaire; *BDP*, beclomethasone dipropionate; *BMI*, body mass index; *CS*, corticosteroid; *Feno*, fraction of exhaled nitric oxide; *FVC*, forced vital capacity; *LABA*, long-acting β_2_-agonist.

∗ Geometric mean (95% CI).

† Median (interquartile range). *Intergroup comparisons* For parametric data, 1-way ANOVA with the Tukey test to compare all pairs of columns: ‡*P* < .05, asthma cluster 1 versus asthma cluster 2; §*P* < .05, asthma cluster 1 versus asthma cluster 3; and ‖*P* < .05, asthma cluster 2 versus asthma cluster 3. For nonparametric data, Kruskal-Wallis test with the Dunn multiple comparison test to compare all pairs of columns: ‡*P* < .05, asthma cluster 1 versus asthma cluster 2; §*P* < .05, asthma cluster 1 versus asthma cluster 3; and ‖*P* < .05, asthma cluster 2 versus asthma cluster 3.

**Table VI:** Quantitative CT indices of asthma phenotypes

	Asthma cluster 1: Severe air trapping, bronchial wall thickening, and bronchial lumen dilatation (n = 11)	Asthma cluster 2: Moderate air trapping (n = 34)	Asthma cluster 3: Severe air trapping and bronchial lumen narrowing (n = 17)	Significance (*P* value)
Proximal airway dimensions (inspiratory)				
RB1 % wall volume	58.1 (2.7)	62.0 (2.6)	64.8 (3.0)	<.005*†‡
RB1 wall area/BSA (mm^2^/m^2^)	25.0 (3.3)	19.2 (2.5)	12.3 (3.2)	<.005*†‡
RB1 lumen area/BSA (mm^2^/m^2^)	18.0 (2.0)	11.7 (1.7)	6.7 (1.7)	<.005*†‡
RB1 total area/BSA (mm^2^/m^2^)	42.9 (4.9)	30.9 (3.8)	18.9 (4.8)	<.005*†‡
RB1 length (mm)	11.6 (2.6)	12.5 (2.5)	13.5 (2.8)	.2
RB1 wall volume (mm^3^)	540.5 (92.8)	453.1 (120.4)	324.2 (102.9)	**<.005**†‡
RB1 wall volume (% greater than upper 95% CI of healthy control subjects)	27	15	6	.3
RB1 LV (mm^3^)	392.2 (78.6)	278.0 (78.0)	176.1 (54.0)	<.005*†‡
RB1 LV (% less than lower 95% CI of healthy control subjects)	9	65	94	**<.005**
RB1 total volume (mm^3^)	932.7 (163.3)	731.2 (194.8)	500.3 (154.4)	<.005*†‡
Pi10 wall area (mm^2^)	16.9 (1.9)	16.2 (1.7)	16.2 (1.4)	.5
Po20 % wall area	64.7 (2.5)	64.8 (2.3)	65.5 (2.5)	.6
Air trapping				
Expiratory VI −850 (%)	23.7 (16.0)	17.2 (11.9)	24.8 (13.9)	.1
Expiratory VI −850 (% greater than upper 95% CI of healthy control subjects)	64	29	59	**.045**
VI_−850/−950_ E-I (%)	−28.6 (12.8)	−31.7 (13.5)	−27.6 (11.4)	.5
VI_−850_ E-I (%)	−35.9 (14.2)	−39.9 (15.5)	−38.3 (12.2)	.7
MLD E/I	0.876 (0.06)	0.857 (0.06)	0.864 (0.05)	.6
MLD E/I (% greater than upper 95% CI of healthy control subjects)	73	53	65	.5
Fractal dimension				
Inspiratory D_av_	1.712 (0.04)	1.686 (0.04)	1.671 (0.04)	**.04**†
Inspiratory D_av_ (% less than lower 95% CI of healthy control subjects)	46	65	77	.2
Expiratory LAC-D −850	1.838 (0.09)	1.814 (0.1)	1.794 (0.05)	.4

Data are expressed as means (SDs). *LAC-D −850*, Fractal dimension of low attenuation cluster at a threshold of −850 HU. *Intergroup comparisons* One-way ANOVA with the Tukey test to compare all pairs of columns: *P < .05, asthma cluster 1 versus asthma cluster 2; †*P* < .05, asthma cluster 1 versus asthma cluster 3; and ‡*P* < .05, asthma cluster 2 versus asthma cluster 3.

## Methods

### CT imaging of study subjects

#### CT acquisition

CT scans were acquired with a multidetector CT scanner, the Siemens Sensation 16, at Glenfield Hospital, Leicester, United Kingdom. All subjects were scanned within 30 minutes of inhalation of 2.5 mg of nebulized salbutamol in the supine position and with shoulders fully abducted to avoid streak artifacts form arm bones. A foam box (LD15; Styrotech Ltd, West Bromwich, United Kingdom) housing 3 electron density rods (LN300, LN450, and “solid water”) from an RMI467 electron density CT phantom (Gammex-RMI Ltd, Nottingham, United Kingdom) was secured over the midpoint on the sternum by using a Velcro belt. The electron density rods LN300, LN450, and “solid water” have electron density relative to water of 0.28, 0.40, and 0.99, respectively. Volumetric whole-lung scans were acquired at full inspiration (near TLC) and at the end of normal expiration (near FRC) in a caudocranial direction to minimize motion artifacts secondary to diaphragmatic motion. All subjects rehearsed inspiratory and expiratory breath holds at least twice before the CT scan. CT scans were acquired with dose modulation switched off at collimation of 16 × 0.75 mm, pitch of 1.5 mm, 120 kVp, 40 mA, 0.5-second rotation time, and scanning field of view (FOV) of 500 mm. Images were reconstructed with a slice thickness of 0.75 mm and a slice interval of 0.5 mm by using a low spatial frequency algorithm (B35f) through a 512 × 512 matrix, with a field of view targeted to include pulmonary areas and a foam box containing 3 electron density rods.

### CT radiation safety

Assessment of radiation exposure caused by research CT scans was performed. The effective dose for full thoracic CT scans was calculated based on Monte Carlo simulations^E1^ of calculated x-ray spectra in an adult, hermaphrodite, mathematic phantom by using the ImPACT CT dosimetry calculator.^E2^ The estimated effective dose for a full thoracic CT scan was 1.5 mSv. The radiation dose associated with common radiologic examinations^E3^ and research CT scans is presented in Table E1.

### Quantitative proximal airway and air-trapping analysis with automated software

Fully automated software, VIDA PW2 software, was used for quantitative airway and densitometric analysis. CT images reconstructed with a low (B35f) spatial frequency algorithm were used.

#### Three-dimensional quantitative airway analysis

The first 5 to 6 generations of the airway tree can be segmented, labeled, and reliably measured by using PW2 software, as described and validated previously.^E4-E7^ Morphologic airway measurements were obtained along each centerline voxel of the lumen perpendicular to the long axis on each airway and averaged over the middle third of the airway segment. LA, total area, and lengths of the right RB1 bronchus and other segmented airways were measured. Average dimensions of 4 (RB1, RB10, LB1+2, and LB10) segmental bronchi in asthmatic patients were also calculated. WA was derived from LA and total area as follows:

WA=Totalarea−LA.

WV, LV, and total volume were calculated by multiplying respective cross-sectional area measurement with airway length. Percentage WV was derived from WV and total volume as follows:

%WV=WV/Totalvolume×100.

The airway measurements, LA, WA, and total area were corrected for BSA.

Airway dimensions of Pi10^E8^ and Po20 were calculated by using regression equations based on airway dimension data for each subject. Internal airway perimeter (Pi) was plotted against the square root of WA for all the measured airways. The WA for a hypothetical airway with a Pi of 10 mm was then determined. Outer airway wall perimeter (Po) was plotted against the square root of WA and the square root of LA for all the measured airways. The WA and LA for a hypothetical airway with a Po of 20 mm was then determined. Po20 percentage WA was determined from Po20 WA and Po20 LA as follows:

Po20%WA=Po20WA/(Po20WA+Po20LA)×100.

#### Quantitative air-trapping analysis

Air-trapping quantification was performed by using whole-lung densitometry of inspiratory (TLC) and expiratory (FRC) CT scans with PW2 software. A threshold-based technique is used to segment the lungs from rest of the thoracic structures and derives densitometric indices from the voxel frequency distribution histogram. Air-trapping indices derived were as follows:

A. *VI at a threshold of −850 HU at FRC.*^E9^ This is defined as the proportion of lung voxels of low density expressed as a percentage of less than a threshold of −850 HU at FRC. A CT density of −850 HU represents the density of a fully distended alveolus. Therefore any voxels less than −850 HU on an expiratory CT scan must represent areas of air trapping.
B. *MLD E/I.*^E10^
C. *VI −850 change on paired inspiratory and expiratory CT scan (VI*_*−850*_ *E-I).*^E11^ The percentage of lung voxels with attenuation values of less than −850 HU was calculated on both inspiratory and expiratory scans. To evaluate the change in VI −850 values, the difference between VI −850 on expiratory CT and inspiratory CT was calculated as follows:

$${\text{VI}}_{-850}\text{E}-\text{I}=\text{VI}-850\;\text{expiratory}\;\text{CT}-\text{VI}-850\;\text{inspiratory}\;\text{CT}\text{.}$$

D. *Voxel index change of percentage voxels between −950 and −850 HU on paired inspiratory and expiratory CT scans (VI_−850/−950_ E-I).*^E11^ The percentage of lung voxels with attenuation values of greater than −950 HU and less than −850 HU were calculated on both inspiratory and expiratory scans. On CT scans, the attenuation threshold of −950 HU has been shown macroscopically^E12^ and microscopically^E13^ to delineate emphysematous areas of the lungs. VI_-850/−950_ was calculated for both inspiratory and expiratory scans by subtracting VI −950 from VI −850. This ensures that any voxels with low attenuation value because of emphysema are eliminated and not included in air-trapping quantification. To evaluate the change in VI_-850/−950_, the difference between VI_-850/−950_ on expiratory CT and inspiratory CT was calculated as follows:

$${\text{VI}}_{-850/-950}\;\text{E}-\text{I}={\text{VI}}_{-850/-950}\;\text{expiratory}\;\text{CT}-{\text{VI}}_{-850/-950}\;\text{inspiratory}\;\text{CT}\text{.}$$

The VI at a threshold of −950 HU and 15th percentile point in HU on inspiratory scans at TLC was also calculated to assess the degree of emphysema, if any.^E12,E13^ The 15th percentile point is defined as a cutoff value in Hounsfield units below which 15% of all lung voxels are distributed. Basic densitometric indices are demonstrated in the line diagram (Fig E1).

All densitometric indices were standardized for extrathoracic air, blood, and 3 electron density rods (Fig E2). Regression equations were calculated from measurements of the densitometric standards (extrathoracic air ventral to patient’s sternum, blood, and 3 electron density rods), with each CT scan of every subject in relation to the standard density measures of the densitometric standards. The regression equations derived were used to adjust all the densitometric indices for each CT scan of every subject. Fig E2 shows derivation of the regression equation for standardization of densitometric indices for one of the subjects.

### Fractal dimension of the airway tree and low attenuation area in lungs

#### Fractal dimension of the airway tree

First, the image of the tracheobronchial tree segmented by using PW2 software was saved as a JPEG file to perform the fractal analysis (Fig E3, *A*). Standard settings were used for each image, with a size of 513 × 518 pixels and resolution of 72 pixels per inch and the subject in normal anatomic orientation. Images were first binarized by using an automated threshold procedure (Fig E3, *B*), in which the pixel color (black or white) for the foreground (segmented tracheobronchial tree) and background was automatically assigned by using ImageJ software. Fractal analysis was performed with the morphologic image analysis software ImageJ plug-in FracLac (version 2.5 Rel. 1e).^E14,E15^ The box-counting algorithm was used to determine the fractal dimension of the binarized image of the segmented tracheobronchial tree for each subject. This algorithm places several grids of decreasing box size over the region of interest (ROI; ie, binarized image of the tracheobronchial tree; Fig E3, *C* and *D*). The number of boxes containing pixels with ROI detail is then counted for each grid, and data are gathered for each box of every grid. The fractal dimension is then expressed as the slope of the regression line for the log-log plot of box size and count.^E15^

The standard FracLac analyses protocol was selected in accordance with the recommendations of the FracLac user manual.^E15^ The size of the series of grids was set to decrease linearly from a maximum box size of 45% of the horizontal ROI size to a minimum size of 1 pixel. Ten global scans were performed for each ROI, with randomly selected starting grid locations to improve the accuracy of the box-counting result. The following measures were derived by using FracLac to describe the tracheobronchial branching in asthmatic and healthy subjects:

A. D_av_: The fractal dimension averaged over 10 global scans that were done at different grid positions.
B. D_sc_: As box size increases relative to image size, the number of boxes required to cover an image stays the same over a long interval of change in size and causes a plateau in the log-log plot of box size and count. The slope-corrected fractal dimension is the fractal dimension corrected for periods of no change in regression data.
C. Most efficient cover fractal dimension (D_e_): The fractal dimension generated from box-counting data in which the box count that required the lowest number of boxes (most efficient cover) for each grid size was used.
D. Slope-corrected most efficient covering fractal dimension (D_sce_): Combination of D_sc_ and D_e_.

#### Fractal dimension of low attenuation areas in lungs

PW2 software was used to calculate the fractal dimension of the low attenuation areas in lungs on inspiratory and expiratory scans. The low attenuation cluster regions on CT scans are contiguous areas of voxels with CT attenuation values of less than a given threshold. Summing the number of voxels in a low attenuation cluster provides the cluster size. The fractal dimension is then expressed as the slope of the regression line for the log-log histogram plot created with low attenuation cluster size and low attenuation cluster number.^E16,E17^ PW2 software calculates the fractal dimension for each lung (right and left) separately. The fractal dimension of low attenuation areas for each subject was expressed as an average of right and left lung fractal dimensions. A threshold CT attenuation value of −950 HU on inspiratory scans and −850 HU on expiratory scans was used to define low attenuation cluster to assess the size and distribution of emphysematous lesions and air-trapping areas, respectively (Fig E4).

### Assessment of the accuracy of airway morphometry performed using PW2 software

A phantom model, the Leicester Airway Phantom described previously,^E18^ consisting of a polystyrene block embedded with 9 cylindrical plastic tubes of varying dimensions, was used. Stereomicroscopy was used to determine the cross-sectional dimensions of the LAP tubes with an accuracy of ±1 μm. A Vernier caliper was used to measure the length of the Leicester Airway Phantom tubes.

CT imaging of Leicester Airway Phantom was performed with Siemens Sensation 16 scanner at Glenfield Hospital, Leicester, United Kingdom, with scanning parameters similar to those of study subjects' scans described above. Images were reconstructed with a slice thickness of 0.75 mm and a slice interval of 0.5 mm by using a low (B35f) spatial frequency algorithm through a 512 × 512 matrix, with a field of view of 350 mm. Three tubes with the smallest dimensions in the Leicester Airway Phantom were not measurable at a reconstruction FOV of 350 mm, and therefore only tubes 4 to 9 were used. The volumetric CT scan of Leicester Airway Phantom tubes of 4 to 9 was analyzed by using the fully automated software VIDA Pulmonary Workstation, version 2.0 (PW2, Vida Diagnostics; http://www.vidadiagnostics.com/]. Morphologic measurements of the phantom tubes were obtained along each centerline voxel of the lumen perpendicular to the long axis on each tube and averaged over the middle third of the tube. LA, total area, and length of 4 to 9 phantom tubes were measured. WA was derived from LA and total area as follows:

WA=Totalarea−LA.

Repeatability analysis for LA, WA, and length measurements of Leicester Airway Phantom tubes 4 to 9 by a single observer 2 months apart showed excellent correlation: LA, ICC = 1 (95% CI, 0.3-1; *P* < .005); WA, ICC = 0.99 (95% CI, 0.3-1; *P* < .005); length, ICC = 1 (95% CI, 0.97-1; *P* < .005; 2-way random-effects model, absolute agreement, and single-measure reliability). Bland-Altman plots for intraobserver repeatability are shown in Fig E5. Measures of LA and WA of Leicester Airway Phantom tubes 4 to 9 made with PW2 software was compared with steromicroscope measures. Length measurements of Leicester Airway Phantom tubes 4 to 9 made with PW2 software were compared with Vernier caliper measures. No significant difference was found when WA/LA measures made with a steromicroscope were compared with measures obtained by using PW2 software (paired *t* test; mean LA, 11.9 [SD, 6.6] vs 11.1 [SD, 6.9], *P* = .06; mean WA, 21.1 [SD, 15.5] vs 20.3 [SD, 11.2], *P* = .7; Fig E6). Mean tube length was underestimated by PW2 software compared with the Vernier caliper (paired *t* test; 48.1 [SD, 8.4] vs 49.4 [SD, 9.0], *P* = .01; Fig E6). Repeatability analysis for LA/WA and length measurements of Leicester Airway Phantom tubes 4 to 9 using a steromicroscope and Vernier caliper, respectively, compared with PW2 measurements showed excellent correlation: LA, ICC = 0.99 (95% CI, 0.82-1; *P* < .005); WA, ICC = 0.95 (95% CI, 0.7-0.99; *P* < .005); length, ICC = 0.99 (95% CI, 0.52-1; *P* < .005; 2-way random-effects model, absolute agreement, and single-measure reliability). Bland-Altman plots of LA and length do not show any systematic bias. The Bland-Altman plot of WA shows that PW2 software overestimates and underestimates dimensions of smaller and larger tubes, respectively, compared with a steromicroscope (Fig E6).

### Principal component and cluster analyses

Principal component and cluster analyses were performed by using quantitative CT data of asthmatic patients. Healthy subjects were not included in this analysis.

#### Principal component analysis

CT data were subjected to unsupervised multivariate modeling by using principal component analysis (orthogonal varimax rotation method) to extract components that best describe the underlying relationship among the quantitative CT variables in all asthmatic patients. Before performing principal component analysis, the suitability of data for analysis was assessed. Inspection of the correlation matrix revealed the presence of many coefficients of 0.3 or greater. The Kaiser-Mayer-Olkin measure^E19,E20^ for sampling adequacy was 0.6, with the recommended value being 0.6 or greater. Results on the Bartlett test of sphericity^E21^ reached statistical significance (*P* < .0005), supporting the factorability of the correlation matrix. Independent components reflecting different asthma CT phenotypes were identified by using principal component analysis of 11 quantitative CT variables that encompassed a broad range of proximal and distal airway measures. The quantitative CT variables used for principal component analysis were as follows: (1) RB1 LA/BSA, (2) RB1 WA/BSA, (3) RB1 total area, (4) RB1 LV, (5) RB1 WV, (6) RB1 percentage WV, (7) expiratory VI −850, (8) MLD E/I, (9) VI change of percentage voxels between −950 and −850 HU on paired inspiratory and expiratory CT scans, (10) expiratory fractal dimension of low attenuation cluster at a threshold of −850 HU, and (11) inspiratory D_av_ of the airway tree. Before performing the principal component and cluster analyses, all variables were Z-normalized. Analysis identified 3 components that contributed to the data set in accordance with the Kaiser criterion^E22^ (eigenvalue >1) and that accounted for 75.2% of the total population variance. Component loading for the selected variables of the 3 independent components are shown in Table E6. Component 1, which accounted for 42.6% of total variance, correlated with RB1 LA/BSA and RB1 LV values and inversely correlated with RB1 percentage WV. Component 2 (23.3% of total variance) correlated with MLD E/I and VI change of percentage voxels between −950 and -850 HU on paired inspiratory and expiratory CT scans. Component 3 (9.3% of total variance) correlated with the expiratory fractal dimension of the low attenuation cluster at a threshold of −850 HU and inspiratory D_av_ of the airway tree. The predominant variable from each component that passed the Kaiser criterion (ie, eigenvalue >1) was selected for statistical cluster analysis.

#### Cluster analysis

After identification of 3 “quantitative CT” components, we used cluster analysis to classify patients with asthma into phenotypes based on quantitative CT indices. The highest loading variable from each component (namely RB1 LA/BSA, MLD E/I, and expiratory fractal dimension of low attenuation cluster at a threshold of −850 HU) was used for cluster analysis. Two steps were involved in statistical cluster analysis. First, hierarchical cluster analysis was performed with the Ward method (using squared Euclidean distance as the interval measure), which generated a dendrogram to determine the number of likely clusters. The period of large change between successive fusion levels in the dendrogram were used to define likely cluster boundaries.^E23^ Three clusters were estimated by using hierarchical cluster analysis of the predominant variable from each component that had been identified by using principal component analysis. Second, k-means cluster analysis was used as the principal clustering technique, with a prespecified number of clusters to determine the cluster membership of asthmatic patients.^E24^
